# Structure-Dependent Effects of Phthalates on Intercellular and Intracellular Communication in Liver Oval Cells

**DOI:** 10.3390/ijms21176069

**Published:** 2020-08-23

**Authors:** Lucie Čtveráčková, Daniel Jančula, Jan Raška, Pavel Babica, Iva Sovadinová

**Affiliations:** 1 RECETOX, Faculty of Science, Masaryk University, Kamenice 753/5, Pavilion A29, 625 00 Brno, Czech Republic; lucie.ctverackova@recetox.muni.cz (L.Č.); raska@med.muni.cz (J.R.); pavel.babica@recetox.muni.cz (P.B.); 2 Department of Experimental Phycology and Ecotoxicology, Institute of Botany of the Czech Academy of Sciences, Lidická 25/27, 602 00 Brno, Czech Republic; sansan@seznam.cz

**Keywords:** gap junctional intercellular communication, gap junctions, hepatotoxicity, MAP-kinases Erk1/2 activation, non-genomic mechanism, oval cells, phthalates, progenitor cells

## Abstract

Humans are exposed to phthalates released from plastics, cosmetics, or food on a daily basis. Phthalates have low acute liver toxicity, but their chronic exposures could induce molecular and cellular effects linked to adverse health outcomes, such as liver tumor promotion or chronic liver diseases. The alternation of gap junctional intercellular communication (GJIC) and MAPK-Erk1/2 pathways in liver progenitor or oval cells can disrupt liver tissue homeostatic mechanisms and affect the development and severity of these adverse outcomes. Our study with 20 different phthalates revealed their structurally dependent effects on liver GJIC and MAPK-Erk1/2 signaling in rat liver WB-F344 cell line with characteristics of liver oval cells. The phthalates with a medium-length side chain (3–6 C) were the most potent dysregulators of GJIC and activators of MAPK-Erk1/2. The effects occurred rapidly, suggesting the activation of non-genomic (non-transcriptional) mechanisms directly by the parental compounds. Short-chain phthalates (1–2 C) did not dysregulate GJIC even after longer exposures and did not activate MAPK-Erk1/2. Longer chain (≥7 C) phthalates, such as DEHP or DINP, moderately activated MAPK-Erk1/2, but inhibited GJIC only after prolonged exposures (>12 h), suggesting that GJIC dysregulation occurs via genomic mechanisms, or (bio)transformation. Overall, medium-chain phthalates rapidly affected the key tissue homeostatic mechanisms in the liver oval cell population via non-genomic pathways, which might contribute to the development of chronic liver toxicity and diseases.

## 1. Introduction

Phthalates, i.e., dialkyl- or alkyl/aryl esters of phthalic acid, represent a group of chemicals used in various consumer products such as building materials, clothing, toys, food packaging, cleaning materials, personal care products, cosmetics, nutritional supplements, medical devices, and others [1]. They have been primarily used as plasticizers and are thus present in various plastic materials, such as polyvinylchloride. Phthalates and their metabolites are ubiquitously occurring in the environment, as numerous studies reported their presence in the air, soil, water, and biota, including human body fluids [2,3,4,5]. Thus, human beings can be exposed to phthalates throughout their lifetime via ingestion, inhalation, dermal, and also intrauterine exposures [6].

The concerns about the adverse health effects led first to a reduction of the most used phthalate for decades, di-(2-ethylhexyl) phthalate (DEHP), but almost simultaneously to an increase in the use of other phthalates [5]. Nowadays, the phthalates which are considered the most toxic, such as DEHP, BBP (benzyl butyl phthalate), DBP (dibutyl phthalate), DOP (dioctyl phthalate), DINP (diisononyl phthalate), and DIDP (diisodecyl phthalate), have also been already regulated for example in the EU, Japan, and the USA [7] (Table S1). Despite these regulations, even in these countries, phthalates are still abundant in products and materials in use, so their environmental occurrence and exposures can be expected for a long time to come [7,8].

The toxicity profiles of phthalates and their potency are determined by their length and branching of dialkyl or alkyl/aryl side chains and physico–chemical properties. In general, phthalates exhibit low acute toxicity in animal models or humans with LD_50_ values being between or above 1–30 g/kg body weight. In sub-chronic rodent studies, phthalates induced dose-related adverse effects in the liver and kidney, and selected phthalates also affected thyroid and testicular tissue [3,6,9]. After chronic exposure, phthalates are known animal carcinogens, classified endocrine-disrupting chemicals, and inducers of liver injury in laboratory animals [10,11]. The human relevance of rodent carcinogenicity of phthalates remains unsettled [9]. For example, DEHP does not exhibit direct genotoxic activity in both rat and human hepatocytes [12]. However, several recent studies suggested links between phthalate exposures and liver inflammation and tumorigenesis in human populations [13,14]. A great deal of concern has been raised about their liver toxicity, particularly due to their intensive detoxification in the liver [13]. U.S. EPA has stated the reference dose of DEHP for the risk of increased liver weight as 20 μg/kg-day for adolescents and women of reproductive age [15]. Collectively, the liver represents an organ directly affected by phthalate exposures and their adverse health outcomes.

The liver is a complex tissue composed of different cell types, where complex and well organized interactions are essential for liver functions and the maintenance of tissue homeostasis in terms of renewal, regeneration, and repair. Hepatocytes are the main cell type of the liver, constituting 50–80% of its mass with specialized vital functions such as protein synthesis, detoxification, and the metabolism of lipids and carbohydrates. Not surprisingly, they are the primary target for hepatotoxic compounds. After the disturbance of their number and function, the cells capable of differentiation into hepatocytes are activated (liver homeostatic regulation). They include non-parenchymal adult liver stem cells and bipotential progenitor cells (LSPCs) [16,17,18], so-called oval cells, and represent the LSPCs invoked during chronic liver injuries [19]. Oval cells possess the characteristics of fetal hepatocytes and biliary cells and can generate hepatocytes and bile duct cells [17]. During the chronic liver injury, the proper function of LSPCs is critical and involves their activation, proliferation, and differentiation to mature hepatocytes and bile duct cells [20]. Thus, the chemically-induced disturbances of LSPC cell regenerative function can disrupt tissue homeostatic mechanisms and contribute to the development and severity of chronic liver toxicities and diseases, including fatty liver diseases, fibrosis, or cirrhosis, and liver cancer [21,22,23,24,25,26,27]. LSPCs likely proliferate at the early stages of carcinogenesis and might give rise to hepatocytes becoming tumor progenitors [17].

A key mechanism for the maintenance of liver tissue homeostasis and functions represents an important route for rapid intercellular signaling, called gap junctional intercellular communication (GJIC) [28]. This communication is mediated through gap junctions connecting adjacent cells leading to direct intercellular interactions in the tissue. Gap junctions provide a network for the trafficking of low molecular mass molecules between the neighboring cells. Its dysfunction has been implicated in a variety of toxicologically and pathologically relevant liver processes, such as cell differentiation and death, inflammation. Its dysregulation in the liver is also associated with prevalent adverse outcomes, such as acute or drug-induced liver injury, chronic cholestatic and fatty liver diseases, fibrosis, cirrhosis, as well as portal hypertension [28,29]. Moreover, the dysregulation of GJIC can promote the growth of transformed cells [30,31,32], and the disruption of liver GJIC and gap junctions is considered to be involved in liver cancer [33,34,35,36]. According to the OECD expert group, the inhibition of gap junction communication is regarded as one of the key hallmarks for the identification of non-genotoxic carcinogens [37].

Many liver toxic compounds and environmental contaminants were reported to induce a rapid and often structure-dependent dysregulation of GJIC in vivo or in vitro [38,39,40,41,42,43,44]. Thus, the dysregulation of GJIC could be the critical cellular process targeted by toxicants via rapid mechanisms independent from genomic signaling, like for example, the activation of various kinases, including mitogen-activated protein kinases MAPK-Erk-1/2 (extracellular signal-regulated kinases 1/2). The MAPK-Erk-1/2 pathway is known to regulate GJIC in different cell types, including liver oval cells [41]. Via the disturbance of liver GJIC, toxicants can cause their disruptive effects on epigenetic regulations, homeostatic processes, and healthy liver functions [35,45]. Previously, there was a structure-dependent relationship of phthalates shown to inhibit intercellular communication in mouse hepatocytes [46].

In this study, we focused on 20 structurally different phthalates representing the most widely used phthalates as well as covering various structural features and molecular weights. Their effects on cell viability, liver GJIC as well as mitogen-activated protein kinases MAPK-Erk1/2 activities in rat liver oval cells WB-F344 were studied. The results presented herein show significant concentration- and time-dependent differences in the ability of structurally different phthalates to rapidly inhibit liver GJIC and activate MAPK-Erk1/2 pathways in liver oval cells. These events might represent critical mechanisms contributing to hazardous liver toxic and hepatocarcinogenic properties of selected phthalates esters.

## 2. Results

### 2.1. Phthalates

Twenty individual phthalates were studied to cover the most used and produced phthalates as well as their different structural features and molecular weights (Table 1 and Figure S1). The selected phthalates include phthalates with mono- as well as di-substituted straight-chain and with short (≤3 C), medium (4–6 C), or long carbon chain length (≥7 C). Their molecular weight ranges from 180 to 447 and logK_ow_ from ~1 to 10.36.

### 2.2. Liver GJIC between Oval Cells in Response to Phthalates

**Figure 1:**
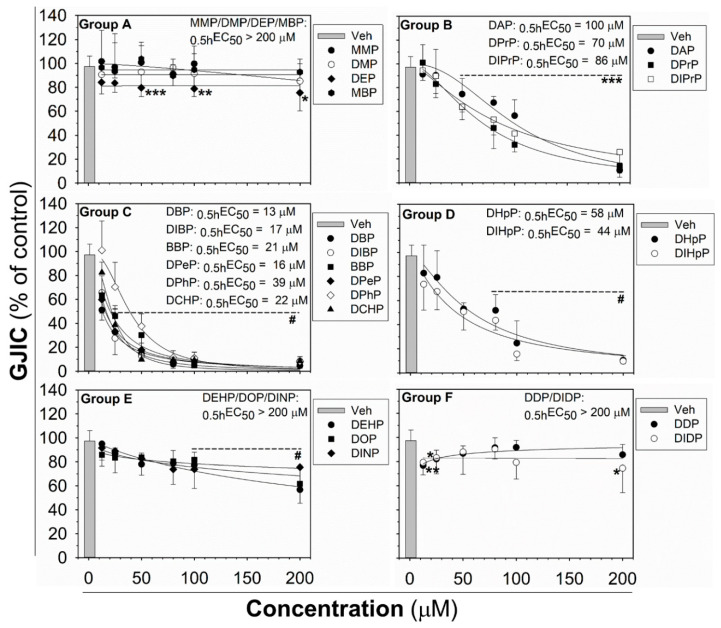
Concentration-dependent effects of phthalates on liver gap junctional intercellular communication (GJIC) in rat oval WB-F344 cells after 0.5 h treatment. The studied phthalates were distinguished into six groups (group **A**–**F**) based on their biological activities, structures, and physico–chemical properties. Data represent the means (SD) of independent experiments (*n* > 3). Significant differences from the vehicle control were determined by one-way ANOVA (*, *p* ≤ 0.050; **, *p* ≤ 0.01; ***, *p* ≤ 0.001) or Kruskal–Wallis ANOVA (#, *p* ≤ 0.05).

**Figure 2:**
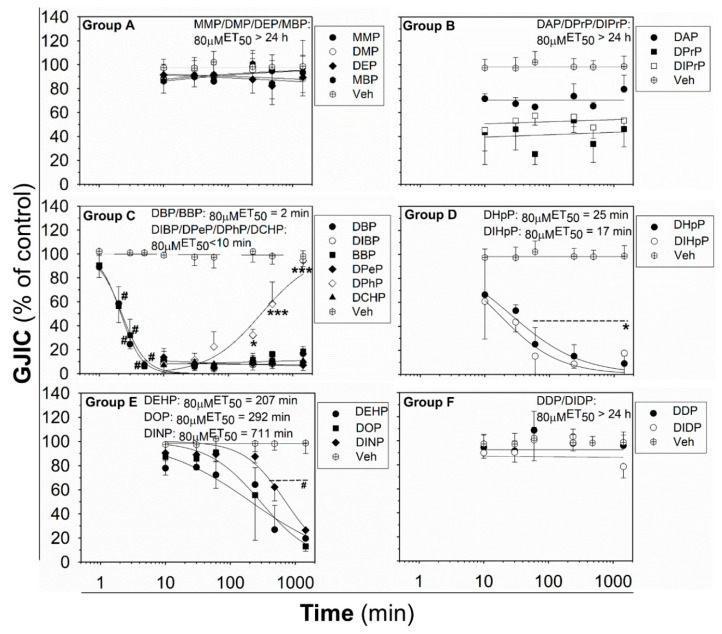
Time-dependent effects of phthalates on liver GJIC in rat oval WB-F344 cells after treatment with a concentration of 80 μM up to 24 h (1440 min). Data represent the means (SD) of independent experiments (*n* > 3). Time points: 1, 2, 3, 5, 10, 30 (0.5 h), 60 (1 h), 240 (4 h), 480 (8 h), and 1440 (24 h) min. The studied phthalates were distinguished into six groups (group **A**–**F**) based on their biological activities, structures, and physico–chemical properties. Significant differences from the effect evaluated after the first exposure time (1 min for DBP, DIB; 10 min for all other phthalates) were determined by one-way ANOVA followed by Dunnett’s test (*, *p* ≤ 0.050; ***, *p* ≤ 0.001) or Kruskal–Wallis ANOVA followed by Dunn’s test (#, *p* ≤ 0.050).

The effects of phthalates on liver oval cells were investigated in immortal rat liver epithelial WB-F344 cells, which exhibit phenotypic characteristics of LSPCs and represent an established in vitro model of oval cells [27,47]. None of the tested phthalates disturbed the integrity and confluency of the cell monolayer up to 24 h exposure to 80 μM concentration, as shown for representative phthalates in Figure S2. Studied phthalates induced structure-, concentration- and time-dependent effects on liver GJIC in oval cells (Figure 1 and Figure 2). From these results, the concentrations causing a 50% decline in GJIC after 0.5 h exposure (_0.5h_EC_50_) and the time required for a 50% decline in GJIC after treatment with 80 μM concentration (_80μM_ET_50_) were calculated (Table 1). Based on their effects on the GJIC, chemical structure, and molecular weight, the studied phthalates can be categorized into six groups—group A–F (Table 1, Figure 1 and Figure 2).

The phthalates from group A (MMP, DMP, DEP, and MBP) are low molecular weight mono- or di-ester phthalates (MW = 180–222 g·mol^−1^) with short or medium side chains. These phthalates have no or only slight effects on GJIC after 0.5 h exposure to concentrations up to 200 μM (_0.5h_EC_50_ > 200 μM; Figure 1 (Group A) and Table 1), or even after longer exposures (up to 24 h) to the concentration of 80 µM (_80μM_ET_50_ > 24 h; Figure 2 (Group A) and Table 1).

The phthalates from group B (DPrP, DIPrP, DAP) have a molecular weight from 246 to 250 g·mol^−1^ and short 3 C side chains. These phthalates induced the rapid and strong inhibition of cell–cell communication at higher concentrations, with _0.5h_EC_50_ values of 70–100 μM (Figure 1 (Group B) and Table 1). GJIC-dysregulating effects caused by the concentration of 80 μM did not become more apparent with increasing exposure time (_80µM_ET_50_ > 24 h; Figure 2 (Group B) and Table 1).

Group C (DBP, DIBP, BBP, DPeP, DCHP, DPhP) represents phthalates with a molecular weight of 278–330 g·mol^−1^ and a medium-sized side chain (4–6 C). Phthalates from this group dysregulated GJIC in WB-F344 cells with the highest potencies, with the _0.5h_EC_50_ values of 13–39 μM (Figure 1 (Group C) and Table 1). At 80 μM concentration, these phthalates rapidly induced the complete inhibition of GJIC within the first 10 min (_80 µM_ET_50_ < 10 min; Figure 2 (Group C) and Table 1). GJIC did not recover during the extended exposure times (up to 24 h), except for DPhP, which had a transient inhibitory effect on GJIC. After 80 μM treatment with DPhP, GJIC was inhibited entirely during the initial 10 min, but then gradually recovered even in the continuous presence of the chemical in the medium, restoring to 50% of the control after 5.5 h of exposure and entirely after 24 h.

The phthalates from group D (DHpP, DIHpP) have a higher molecular weight of 363 g·mol^−1^ and bear a longer 7 C-side chain. These phthalates had a medium effect on liver GJIC between oval cells with a _0.5h_EC_50_ value of ~50 µM (Figure 1 (Group D) and Table 1) with a relatively rapid response (_80μM_ET_50_~20 min; Figure 2 (Group D) and Table 1).

DEHP, DOP, and DINP, which were categorized into group E, have a molecular weight of 391–419 g·mol^−1^ and long side chains (7–9 C). They caused only a weak GJIC inhibition after 0.5 h exposure (down to 60–80% of the control) with _0.5h_EC_50_ > 200 μM (Figure 1 (Group E) and Table 1). With increasing exposure time, their effects on GJIC became more pronounced, with the _80μM_ET_50_ values ranging between 3.5 and 12 h, and the nearly complete inhibition of GJIC was observed after 24 h (Figure 2 (Group E) and Table 1).

Finally, the phthalates with the highest molecular weight of 447 g·mol^−1^ (DDP, DIDP) and the longest chain length (C10) belong to group F. These phthalates had effects similar to group A, i.e., no or only slight effects on liver GJIC with _0.5h_EC_50_ > 200 μM (Figure 1 (Group F) and Table 1) not increasing with exposure time (_80 µM_ET_50_ > 24 h; Figure 2 (Group F) and Table 1).

### 2.3. Viability of Liver Oval Cells in Response to Phthalates

**Figure 3:**
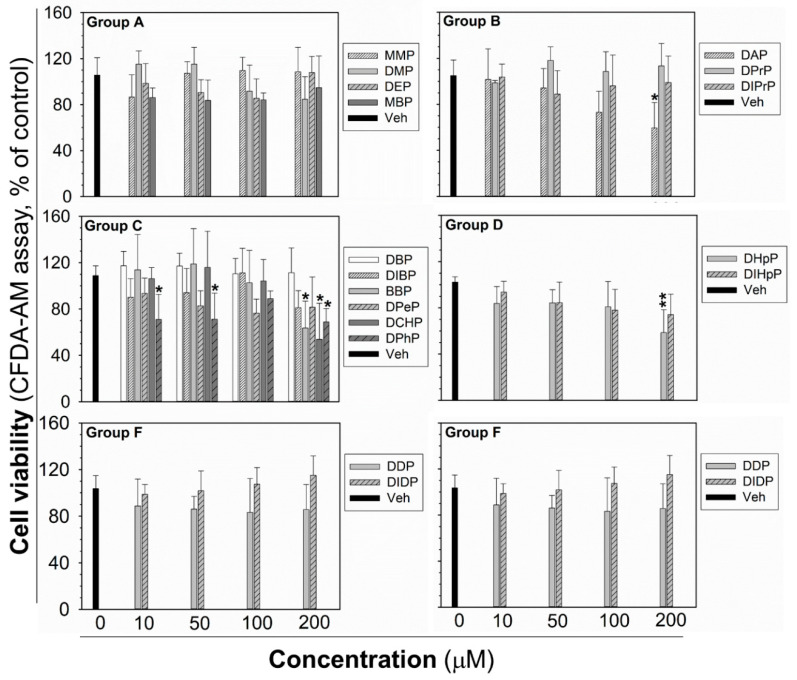
Cell viability of rat liver oval WB-F344 cells after 24 h treatment with phthalates evaluated by CFDA-AM assay. The studied phthalates were distinguished into six groups (group **A**–**F**) based on their biological activities, structures, and physico–chemical properties. Data represent the means (SD) of independent experiments (*n* > 3). Significant differences from the vehicle control were determined by one-way ANOVA followed by Dunnett’s test (*, *p* ≤ 0.050; **, *p* ≤ 0.01). CFDA-AM—5-carboxyfluorescein diacetate-acetoxymethyl ester.

The cytotoxicity testing was done for each phthalate to establish the highest non-cytotoxic concentration after 24 h exposure, to be sure that the observed GJIC inhibition was not due to cytotoxicity and decreased cell density by necrotic or apoptotic cell death, or inhibited proliferation. After 24 h exposure, the cytotoxicity of phthalates (10–200 µM) was assessed by the simultaneous evaluation of three vital cellular processes: cell respiration and dehydrogenase activity by Alamar Blue^®^ reduction, esterase activity and membrane integrity by CFDA-AM (5-carboxyfluorescein diacetate-acetoxymethyl ester) cleavage and retention, and lysosomal membrane integrity and energetic imbalance by neutral red uptake (NRU). None of the phthalates induced lethal cytotoxicity or a reduction of cell viability, according to Alamar Blue^®^ assay (Figure S3). Similarly, no significant effects on NRU were observed, except for DCHP, causing a partial reduction of dye uptake (by an app. 50%) at the highest experimental concentration (Figure S4). CFDA-AM cleavage was the most sensitive parameter (Figure 3), which was partially, but significantly reduced (by app. 30–50%) by DAP, BBP, DCHP, DPhP, and DHpP at 200 µM concentration, i.e., the concentration two- to ten-times higher than their _0.5h_EC_50_ values for GJIC inhibition. DPhP caused no concentration-dependent effect on cell viability in the CFDA-AM assay, since it reduced cell viability at the same extent (app. 30% decrease) at the concentrations of 10 and 50 μM, but not at the concentration of 100 μM (90% of non-treated control).

In addition, the eventual effects of phthalates on cell density, attachment, and confluency of the monolayer were observed by brightfield microscopy in each SL/DT (scalpel loading/dye transfer) experiment. None of the tested phthalates disturbed integrity and 100% confluency of the cell monolayer up to 24 h exposure to 80 μM concentration, as shown for the representative phthalates (Figure S2). Moreover, the intact plasma membrane of healthy cells has low permeability for lucifer yellow. In contrast, the occurrence of damaged and necrotic cells in the SL/DT assay leads to more intense lucifer yellow staining in the background, i.e., outside the cut and dye-transfer area [48], as demonstrated for a cytotoxic treatment of WB-F344 cells with 100 μM triclosan (Figure S5). No such signs of cell damage or cytotoxicity were observed for any of the tested phthalates up to 24 h exposure to 80 μM, including the phthalates that were found to reduce cell viability at higher concentrations by more than 25% (25–50%; DAP, DIBP, BBP, DPeP, DCHP, DPhP, DHpP, DIHpP) in at least one of the cell viability assays (representative images shown in Figure S5).

### 2.4. MAPK-Erk1/2 Pathway in Liver Oval Cells in Response to Phthalates

**Figure 4:**
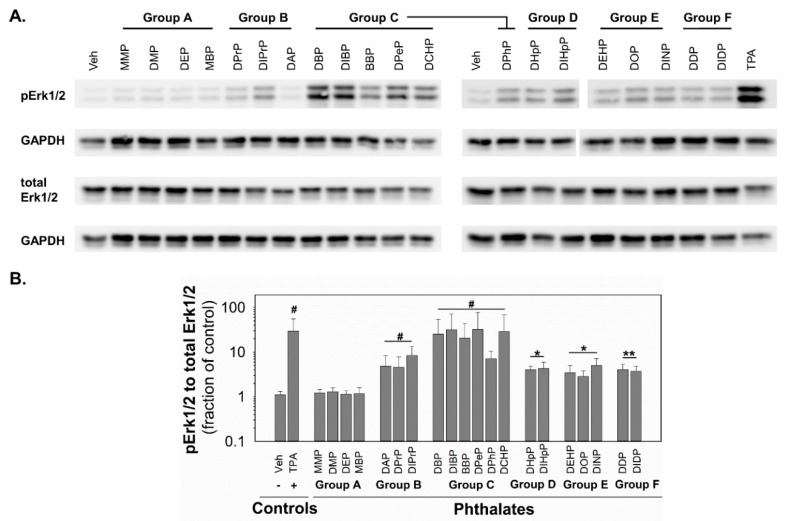
Activation of MAPK-Erk1/2 pathway in rat liver oval WB F-344 cells in response to 0.5 h treatment with phthalates (80 µM). (**A**) A representative immunoblot for phosphorylated Erk1/2 (pErk1/2), total Erk1/2, and GAPDH (glyceraldehyde-3-phosphate dehydrogenase). The studied phthalates were distinguished into six groups (group A–F) based on their biological activities, structures, and physico–chemical properties. The positive control was treated with 10 nM TPA (12-O-tetradecanoylphorbol 13-acetate). (**B**) Densitometric analysis of pErk1/2 normalized to total Erk1/2 represented as the fraction of the control. Data are presented as the means (SD) of independent experiments (*n* > 3), significant differences from the vehicle control were determined by two-tailed *t*-test (*, *p* ≤ 0.050; **, *p* ≤ 0.010) or Mann–Whitney U test (#, *p* ≤ 0.05).

Since MAPKs and their pathways are crucial for healthy liver function [49] and implicated in the control of GJIC [45], the changes in the activity of MAPK-Erk1/2 were investigated in response to phthalates in rat liver oval WB-F344 cells. After 0.5 h exposure of WB-F344 cells to 10 nM TPA (12-O-tetradecanoylphorbol 13-acetate), which was used as a positive control and known GJIC inhibitor and tumor promoter, rapidly and significantly increased (~30-fold increase) the levels of phosphorylated, i.e., activated, MAPK-Erk1 and 2 (Figure 4; original, uncropped and unadjusted images in Figures S6–S9). The phthalates from group A did not induce any detectable phosphorylation of MAPK-Erk1/2. All the phthalates from the other groups activated the MAPK-Erk1/2 pathways with different potencies (Figure 4 and Table 1). The phthalates from groups D–F were weak activators and induced MAPK-Erk1/2 phosphorylation to levels 3–5-fold above the control. The phthalates from group B were medium activators of MAPK-Erk1/2 with 5–8-fold higher levels of phosphorylation than in the vehicle-treated cells. The most potent activators were shown to be the phthalates from group C, which increased levels of phosphorylated MAPK-Erk1/2 to 7–32-fold of that of the control.

### 2.5. Expression of Peroxisome Proliferator-Activated Receptors (Ppar) in Rat Liver Oval Cells WB-F344

**Figure 5:**
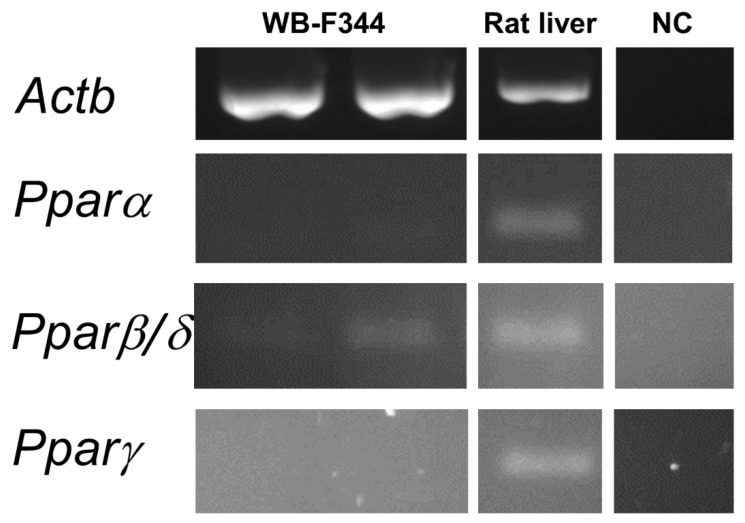
Expression of the *Ppar* isoforms in rat liver oval WB-F344 cells. RNA was isolated independently from two different cultures of WB-F344. Expression of *Pparα*, *ß/δ*, and *γ* was determined by RT–PCR. The reference gene β-actin (*Actb*) was used as a loading control. A sample of RNA extracted from rat liver tissue was used as a positive control to confirm the successful amplification of all *Ppar* isoforms by RT-PCR. Negative control (NC) sample was negative for all isoforms.

The hepatotoxic effect of phthalates via Pparα signaling networks have been observed in rodents, but not in humans [50,51]. To find out if the observed effects caused by phthalates on liver GJIC or MAPK-Erk 1/2 signaling pathways in oval cells in this study might be connected with Ppars, we evaluated the expression of Ppar isoforms in WB-F344 cells. The WB-F344 cells under the conditions used in this study did not express *Pparα* and *Pparγ*, while *Pparβ*/*δ* was expressed only at a low level, as measured by RT-PCR (Figure 5; original, uncropped and unadjusted images in Figures S10 and S11). Therefore, the effects of phthalates on the model of WB-F344 cells and Ppar signaling networks are not likely to be related.

## 3. Discussion

Even though phthalates are nowadays regulated in some countries, hundreds of tons are still produced all around the world, and products containing phthalates are still broadly used, leading to ongoing everyday life human exposures. Animal studies suggested that some phthalates cause hepatotoxicity, developmental, and reproductive toxicity [52] (Table S2). Human phthalate exposures were associated with the increased risks of metabolic disorders, including obesity, diabetes, insulin resistance, and deteriorated liver function [53,54,55]. Chronic exposures can lead to the continuous accumulation and intensive detoxification of phthalates in the liver [56]. Thus, liver cells are likely to be exposed to the highest concentration of phthalates in the body [57], and it is crucial to understand the liver toxicity of phthalates.

In our study, the phthalates were discovered as potent dysregulators of GJIC and activators of the MAPK-Erk1/2 pathways in liver oval cells, and these effects were structure-dependent. Because of their critical role in supporting liver homeostasis, liver gap junctions and GJIC are known to be affected in chronic liver diseases and toxicities [34,58]. However, information on the effect of phthalates on liver GJIC is relatively scarce, and the data are mostly from tests in vivo or with hepatocytes (Table S2). Previous studies found that DEHP and DINP inhibited GJIC in vivo in the liver of mice and rats, but not in the liver of hamsters or monkeys [59,60,61]. These in vivo effects were primarily attributed to major biotransformation products of DEHP and DINP, namely mono(2-ethyl hexyl)phthalate (MEHP) and mono(isononyl)phthalate (MINP). These metabolites and other phthalate monoesters with longer (>7 C) or branched side chains also inhibited GJIC in vitro in primary mouse or rat hepatocytes. However, they did not affect GJIC in the hamster, monkey, or human hepatocytes after 4–24 h exposures to 200–500 µM. This species-specific pattern was attributed to intraspecific differences in the effects of phthalate monoesters on peroxisomal proliferation (thus on the activation of Pparα and its signaling networks). The human relevance of rodent data on phthalate-induced tumor promotion, peroxisome proliferation, and GJIC inhibition is questionable [50,62,63].

In comparison with the earlier data reported for longer/branched monoesters in primary mouse or rat hepatocytes, we observed qualitatively and quantitatively different responses in rat liver oval cells to phthalic acid diesters. Most notably, the phthalates with medium-length (4–6 C) carbon side chains such as DBP, DIBP, BBP, DPeP, DCHP, and DPhP (i.e., Group C) were the most active and rapid GJIC inhibitors with effective _0.5h_EC_50_ values of 13–39 μM (4–12 mg/L), as well as the most potent MAPK-Erk1/2 activators. The effective concentrations and times required for GJIC inhibition by these phthalate diesters were lower than those previously reported for longer/branched-chain monoesters in primary mouse and rat hepatocytes, where concentrations >50–100 μM were usually required to induce ≥50% inhibition of GJIC after 4–24 h of exposure [46,64]. According to these earlier studies in rodent hepatocytes (other types of liver cells were not assessed regarding the possible liver toxicity of phthalates), phthalate monoesters with shorter straight chains did not inhibit liver GJIC [46]. In contrast, monoesters with longer/branched-chains inhibited GJIC with a relatively uniform concentration- and time-response pattern [64].

Here, we were able to separate the tested phthalates into six different groups with a distinct profile of liver GJIC inhibition and MAPK-Erk1/2 activations. Short-chain diesters and monoesters (Group A) neither inhibited GJIC nor activated MAPK-Erk1/2. Phthalate diesters with 3 C (Group B) or 7 C (Group D) side chains were also rapid GJIC inhibitors and MAPK-Erk1/2 activators. However, still less potent than Group C. Interestingly, the effects of Group B phthalates (DPrP, DIPrP, DAP) did not become more pronounced with increasing exposure time, as observed for Group D (DHpP, DIHpP). The phthalates from group E (DEHP, DOP, DINP) rapidly activated MAPK-Erk1/2 but caused only a weak inhibition of GJIC even at concentrations ~200 μM. Nevertheless, their GJIC inhibiting effects progressed with longer exposure times (_80μM_ET_50_ values of 3.5 to 12 h). Finally, long-side chain phthalate diesters (Group F—DIDP, DDP) did not induce major effects on GJIC like Group A. However, in contrast to Group A, they rapidly activated MAPK-Erk1/2 with a potency comparable to Group B, D or E. The alterations of GJIC by Groups B–D and the activation of MAPK-Erk1/2 by Groups B–F were observed within several minutes of exposure, indicating they were most likely caused directly by the parental phthalate diester compounds, not by monoesters produced by eventual phthalate (bio)transformation.

We were able to observe relatively more nuanced structure-dependent effects of phthalate diesters in our study than in the previous studies of GJIC in primary hepatocytes exposed to phthalate monoesters. Importantly, similar structure-dependent activity has been observed in the studies reporting adverse developmental effects after treatment with phthalates. Short phthalates (methyl and ethyl) exhibit low or no rodent developmental toxicity, while medium-size phthalates (butyl) increased developmental malformations [51,52]. In agreement with this structure-dependent activity, the phthalates with medium carbon side chains were also found to be the most active in ToxCast assays with the percentage of “active” assays ranging from 7.33% to 26.97% (Table S2). These phthalates were also the most active in ToxCast assay endpoints in each gene set concerning hepatotoxicity or hepatic tumors and in the hepatotoxicity assays conducting not only with rat hepatocytes but also with human hepatocytes or human liver HepG2 cells [65].

For phthalates with medium- or long-side chain length, the transcriptional activation of Pparα, and subsequent downstream events such as peroxisome proliferation, represent essential mechanisms of toxicity in rodents [50,51]. This finding was confirmed for active phthalates from this study in appropriate ToxCast assays (Table S2). Peroxisomal proliferation is a species-specific process where humans are considered to be a largely non-responsive species to this mechanism of action [57,66], due to qualitative differences in the transcriptional networks controlled by Pparα activation [50]. However, it has also been shown that PPARα activation and peroxisomal proliferation are not essential for liver tumorigenesis induced by phthalates in humans [10,67]. Combined data from animal models and exposed humans suggest that multiple molecular signals and pathways activated in several cell types in the liver, rather than a single molecular event in hepatocytes, contribute to the carcinogenic effects of DEHP [68].

Our results support that the Pparα-independent events induced in the critical population of liver oval cells might participate in liver toxicity and liver tumorigenesis caused by phthalates because Pparα was not found to be significantly expressed in WB-F344 cells. The inhibition of GJIC and the activation of MAPK in these liver oval cells by active phthalates were therefore elicited by another, PPARα- and peroxisome proliferation-independent mechanism. Moreover, the timing of oval cell responses to Group B–D phthalates (≤30 min) further suggest that these effects were not mediated via the transactivation of nuclear receptors, such as Pparα, and subsequent gene expression changes (i.e., genomic mode of action), but rather through rapid alterations of signal transduction pathways controlling the gating of gap junction channels or the activation of MAPKs (i.e., non-genomic mechanisms). However, genomic mechanisms, other than the Ppar signaling pathway, possibly in combination with phthalate transformation to monoesters, could be involved in the delayed inhibition of GJIC induced by Group E or in the later phases of sustained GJIC inhibition caused by Groups C or D.

Phosphorylated MAPK-Erk 1/2 (pErk1/2) is the activated form of MAPK-Erk, and the key component of the Ras/Raf/Mek/Erk pathway, whose balanced liver activity is crucial for healthy liver function [49]. The role of MAPK-Erk1/in hepatic metabolism and its increase in states of obesity have also been discussed [49]. Phosphorylated MAPK-Erk1/2 activates a variety of target molecules involved in the regulation of cell cycle progression, proliferation, apoptosis, and migration of liver cells, and also other processes crucial to promote the development of liver cancer, such as invasion and metastasis [69]. In our study, all phthalates except Group A rapidly activated the MAPK-Erk1/2 pathways in WB F-344 cells. Similar to a known tumor promotor, TPA, MAPK-Erk1/2 activation was associated with the rapid (Groups B–D) or delayed (Group E) inhibition of GJIC. Thus, this rapid MAPK-Erk 1/2 activation in liver oval cells after phthalate treatment might lead to an imbalance of hepatic tissue homeostasis and other liver functions, as well as might contribute to tumor-promoting and progressing stages of hepatocellular carcinoma. The activation of liver oval cells has been associated with liver tissue damage, inflammation, fatty liver disease, and these cells were found to play a crucial role in the progression of hepatocellular carcinoma [26]. Correspondingly, BBP, inhibiting GJIC and activating MAPK-Erk1/2 in our study, was previously found to induce invasion, migration, and the angiogenesis of hepatocellular carcinoma cells Huh7 via rapid non-genomic aryl hydrocarbon receptor/G-protein dependent mechanism [70].

In our study, the alterations of rapid signaling events were observed in WB-F344 cells, which have characteristics of liver oval cells or LSPCs. These types of cells are activated during various liver (toxico-)pathologies, and their activation correlates with the levels of tissue damage and inflammation [21,26]; therefore, they are crucial for liver healing and regeneration. Specifically, WB-F344 cells are non-tumorigenic GJIC-communicating rat liver oval cells that have been shown to undergo in vitro transformation into malignant/tumorigenic and GJIC-deficient cells in response to oncogene overexpression [71], mutagenization [72] as well as chemical exposures [73,74].

Our findings indicate the importance of studying the effects of chemicals on GJIC and rapid signaling mechanisms not only in differentiated hepatocytes, where the major building block of gap junctions is connexin 32 [29,34,58] but also in liver oval cells and LSPCs, such as WB-F344, which predominantly express connexin 43 [27]. Connexin 43 is the most abundant and studied connexin type, whose activity and function can be regulated by the direct phosphorylation caused by many kinases, including MAPK-Erk-1/2 [39,41,75]. While the activation of MAPK kinases is considered to be one of the mechanisms of GJIC dysregulation [41,76,77], it has been reported that various environmental toxicants and endocrine disrupters can dysregulate GJIC via mechanisms independent from the MAPK-Erk1/2 pathway, even when MAPK-Erk1/2 activation is associated with GJIC dysregulation [41,43,44,45]. Although GJIC-inhibiting phthalates also activated the MAPK-Erk1/2 pathways in our study, the rapid MAPK-Erk1/2 activation caused by phthalates from Groups E and F was not necessarily associated with the rapid inhibition of GJIC. It suggests that other mechanisms and pathways, independent or co-dependent on pErk1/2, were probably also involved in the rapid dysregulation of GJIC in response to phthalates. The upstream regulators of GJIC and Erk1/2 targeted by phthalates in connexin 43-expressing liver oval cells and the exact signal transduction mechanisms leading to GJIC inhibition thus need to be elucidated in future studies.

The effective concentrations of the most potent phthalates from Group C were ~4–25 mg/L, which are concentrations very close to the concentrations measured in the human biomonitoring of phthalates and their mixtures. The monitoring is predominantly based on the detection of phthalate metabolites in urine, which represents the main route of phthalate elimination. However, phthalates and their metabolites can also be detected in other body fluids, including blood (plasma and serum), amniotic fluid, breast milk, saliva, or seminal fluid [78]. The concentrations of selected phthalate diesters (DEP, DBP, BBP, DEHP) in human blood plasma or serum can be often found in the range of 0.001–0.5 mg/L [79,80,81,82,83,84], with even higher mean values (0.2–4.4 mg/L) reported for specific populations, such as Asian women in advanced stages of endometriosis [82,83,84].

## 4. Materials and Methods

### 4.1. Chemicals

All ingredients for phosphate-buffered saline (PBS, 10 mM, pH 7.4), formaldehyde, methanol, phthalates (purity: >97%), lucifer yellow dye dilithium salt, 12-O-tetradecanoylphorbol 13-acetate (TPA) and neutral red solution were obtained from Sigma-Aldrich (St. Louis, MO, USA). Additionally, 5-carboxyfluorescein diacetate-acetoxymethyl ester (CFDA-AM) and Alamar Blue^®^ solution were purchased from Thermo Fisher Scientific (Waltham, MA, USA).

### 4.2. Cell Culture and Experimental Set-up

Rat liver epithelial cells WB-F344 [47] were kindly provided by Prof. Trosko and Dr. Upham (Michigan State University, the College of Human Medicine, Department of Pediatrics and Human Development, MI, USA). The cells were cultured in DMEM media (Thermo, Cat. No. 11880) supplemented with 2 mM glutamine (GE Healthcare, Chicago, IL, USA) and 5% fetal bovine serum (Biosera, Nuaille, France), grown in 25 cm^2^ tissue culture flasks (TPP, Trasadingen, Switzerland) at 37 °C in a humidified atmosphere containing 5% CO_2_ and passaged 2–3× per week. Before each experiment, the WB-F344 cells were seeded (40 × 10^3^ or 20 × 10^3^ cells/cm^2^) in a 96-well microplate (TPP; cell viability assay) or a 35 mm-dish (TPP; GJIC assay and Western blot) and cultured for 48 h (cell seeding density: 40 × 10^3^ cm^2^) or 72 h (cell seeding density: 20 × 10^3^ cm^2^) to reach 100% confluence. Phthalates were dissolved in acetonitrile and TPA in ethanol. Each dissolved compound was added directly to the culture media. Vehicle concentration did not exceed 1% (*v*/*v*) in any experiment, and the corresponding vehicle or negative (non-treated) controls were conducted in each experiment.

### 4.3. Cell Viability Assay

After 24 h treatment, the exposed cells were rinsed with PBS (pH 7.2) and incubated in serum-free culture media containing 5% *v*/*v* Alamar Blue^®^ and 4 µM CFDA-AM. After a 30 min incubation, the fluorescence was measured using microplate reader Synergy 4 Reader (BioTek, Winooski, VT, USA) at 485/520 nm excitation/emission for CFDA and 530/590 nm excitation/emission for Alamar Blue^®^. The cells were then rinsed with PBS and incubated for 2 h with 50 µg/mL of neutral red dissolved in serum-free culture media and washed again with PBS. Accumulated neutral red was extracted with 50% (*v*/*v*) ethanol-1% (*v*/*v*) acetic acid and quantified spectrophotometrically (Synergy 4) at 540 nm with 690 nm reference wavelength. Fluorescence and absorbance readings from the assay blank wells without cells were subtracted from the experimental wells before data analysis.

### 4.4. GJIC Assay

GJIC was evaluated by the scalpel load/dye transfer (SL/DT) technique adapted after the method of [85] and modified according to the recent protocols [42,86]. The exposed cells were rinsed with PBS supplemented with calcium and magnesium (CaMgPBS; pH 7.2). Fluorescent dye lucifer yellow (1 mg/mL, dissolved in CaMgPBS) was added to the cells, and three parallel cuts with a surgical scalpel blade were done per dish to introduce the dye into the cell monolayer. After 3 min of incubation to allow the diffusion of lucifer yellow across the monolayer through functional GJ, the cells were rinsed with CaMgPBS and fixated with a 4% (*v*/*v*) formaldehyde solution in PBS. Dye-transfer, which is proportional to GJIC, was documented by fluorescence microscope Axio Observer.Z1 equipped with a 10× objective, AxioCam HRc camera, Filter Set 05 (AF 430 channel: excitation-390–440 nm, emission: 470 LP) and AxioVision software (Carl Zeiss Microscopy, Jena, Germany). A representative image was taken from each cut, and dye transfer was evaluated as an area of lucifer yellow-stained cells along with each cut by ImageJ [87]. The fluorescence area from the positive control treated with a model GJIC dysregulator, TPA [77], at a dose that induced the complete inhibition of GJIC, was subtracted from each treatment to calculate the net dye-transfer area. In addition, the exposed cells were evaluated by brightfield microscopy for signs of cell detachment, disruptions of 100% confluency and of the integrity of cell monolayer, which can affect the results of GJIC assay.

### 4.5. Western Blot

Western blot was done according to [88]. Briefly, after 0.5 h exposure, the cells were rinsed with PBS and lysed with 4% (*w*/*v*) sodium dodecyl sulfate (SDS) in 20 mM Tris-HCl with 1 mM dithiothreitol. Total proteins were quantified by the DC Protein Assay (Bio-Rad, Hercules, CA, USA). Proteins (10 µg per sample) were separated using SDS-PAGE on 10% gel, followed by Western transfer to Immobilon-P membrane (Millipore, Darmstadt, Germany). Immunodetection was conducted by following primary antibodies: 1:1000 diluted rabbit anti-p44/42 (Cell Signaling, Danvers, MA, USA; #4695S) to detect total Erk1/2 and anti-phospho-p44/42 (Cell Signaling; #4370S) to detect phosphorylated (activated) Erk1/2, and mouse antibody anti-GAPDH (anti-glyceraldehyde-3-phosphate dehydrogenase; Millipore, #MAB374) to detect a reference protein. After washing the membranes, 1:2500 diluted anti-rabbit (Cell Signaling, #7074S) or anti-mouse (Cell Signaling, #7076S) antibodies labeled with horseradish peroxidase were used to detect primary antibodies. Immunoblots were developed using Clarity ECL Western Blotting Substrate (Bio-Rad) and chemiluminescence documented by the gel imaging system MF-Chemibis (DNR Bio-Imaging Systems, Neve Yamin, Israel). Band densities were determined by ImageJ [87], and the density of the activated (phosphorylated) Erk1/2 bands was normalized to the density of the total Erk1/2 band. TPA at the concentration of 10 nM was used as a model activator of a MAPK-Erk1/2 pathway [77].

### 4.6. Reverse Transcription-Polymerase Chain Reaction (RT-PCR)

RT-PCR was done according to [88]. RNeasy Plus Mini (QIAGEN, Hilden, Germany) was used to isolate the total RNA from the cells. Transcriptor First Strand cDNA Synthesis Kit (Roche, Basel, Switzerland) was used to prepare the cDNA, and Phusion High-Fidelity DNA Polymerase kit (ThermoFisher) to conduct PCR. The primers (Table S3) were designed by Primer3 (version 4.1.0) [89] to bind on an exon–intron border or different exons to minimize the inaccuracies due to possible genomic contamination, to recognize all known transcript variants of a target gene and to avoid pseudogene counterparts. Primary lysates from adult rat liver were kindly provided by the Department of Pathological Physiology (Masaryk University, Brno, Czech Republic). They were used as positive controls to detect mRNA of *Ppar*s.

### 4.7. Data and Statistical Analyses

Appropriate vehicle control was included in all experiments, and vehicle treatments did not significantly differ from the non-treated cells (negative control). All quantitative data (GJIC assay, cell viability assay, Western blot densitometry) were normalized to the negative or vehicle controls from the corresponding experiment and reported as the percentage of the control (negative control = 100%), or fraction of the control (FOC, negative control = 1). Control-normalized data from at least three-times independently repeated experiments were combined for all statistical analyses and data presentation. Curve-fitting was done using a non-linear, 4-parameter sigmoidal regression model using GraphPad Prism 5 (GraphPad Software, La Jolla, CA, USA). Statistical analysis was done using SigmaPlot 12.1 (Systat Software GmbH, Erkrath, Germany). Data passing normality (Shapiro–Wilk’s test) and equal variance tests were compared by *t*-test or by one-way ANOVA followed by Dunnett’s test. Data with unequal variances or non-normal distribution were evaluated by the Mann–Whitney U test, or by the Kruskal–Wallis ANOVA on ranks followed by Dunn’s test. *p*-values lower than 0.05 were considered as statistically significant.

## 5. Conclusions

Altogether, toxicologically relevant concentrations of phthalate diesters with medium length side chains elicited rapid and likely Pparα-independent signaling events in rat liver oval WB-F344 cells. This type of liver cells is increasingly recognized for its role in tissue homeostasis, chronic toxicities, and diseases, including hepatocellular carcinomas [17,19,21]. While liver-toxic and hepatocarcinogenic effects of phthalates are continuously discussed [65], most studies are focused on genomic signaling as the principal mechanism, and considering hepatocytes or hepatoma cell lines as the target cell populations involved in phthalate tumor-promoting and liver-toxic effects (Table S2). However, our results suggest that phthalates can also act via non-genomic mechanisms in liver oval cells or LSPCs. The GJIC and MAPK-Erk1/2 signaling pathway represent key mechanisms involved in the maintenance of liver tissue homeostasis, whose disruption in the critical population of liver oval cells might be contributing to phthalate-induced hepatotoxicities and the possible development of chronic liver diseases such as liver cancer. These mechanisms and cellular models should be further addressed by future studies to improve phthalate hazard and risk assessment concerning the ongoing occurrence of phthalates in everyday life. GJIC represents an interesting cellular endpoint, which can be nowadays assessed in vitro in a (semi)high-throughput set-ups, and thus can be utilized for the non-genotoxic carcinogens assessment, chemical hazard identification as well as mechanistic studies [48,90,91].

## Figures and Tables

**Table 1:** Overview of the studied phthalates and their structures, physico–chemical properties, and biological activities, based on which they were distinguished into six groups—group A–F. The color scale is green to yellow to red with higher effects getting the green color and low or no effects getting the red color.

Phthalate	Carbon Chain Length	MW ^a^ g/mol	Log Kow ^a^	Group	GJIC		MAPK-Erk1/2 Activation
					_0.5h_EC_50_^b^ (µM)	_80μM_ET_50_^c^ (min)	FOC ^d^ (0.5 h)
**MMP** Monomethyl phthalate	Short	180	9 × 10^−1^ to 1.50	A	>200	>1440	0
**DMP** Dimethyl phthalate	Short	194	1.46 to 1.90	A	>200	>1440	0
**DEP** Diethyl phthalate	Short	222	2.21 to 3	A	>200	>1440	0
**MBP** Monobutyl phthalate	Medium	222	2.37 to 3.07	A	>200	>1440	0
**DPrP** Dipropyl phthalate	Short	250	3.14 to 3.87	B	70	>1440	5
**DIPrP** Diisopropyl phthalate	Short	250	2.61 to 3.48	B	86	>1440	8
**DAP** Diallyl phthalate	Short	246	2.92 to 3.36	B	100	>1440	5
**DBP** Dibutyl phthalate	Medium	278	4.39 to 4.83	C	13	2	24
**DIBP** Diisibutyl phthalate	Medium	278	3.81 to 4.46	C	17	10	32
**BBP** Benzyl butyl phthalate	Medium	312	3.57 to 4.91	C	21	2	21
**DPeP** Dipentyl phthalate	Medium	306	5.19 to 5.89	C	16	10	32
**DCHP** Dicyclohexyl phthalate	Medium	330	4.79 to 6.20	C	22	10	28
**DPhP** Diphenyl phthalate	Medium	318	2.82 to 4.61	C	39	10	7
**DHpP** Diheptyl phthalate	Long	363	5.65–6.82	D	58	25	4
**DIHpP** Diisoheptyl phthalate	Long	363	7.4	D	44	17	4
**DEHP** Di-(2-ethylhexyl) phthalate	Long	391	5.11–8.35	E	>200	207	3
**DOP** Dioctyl phthalate	Long	391	7.84 to 9.08	E	>200	292	3
**DINP** Diisononyl phthalate	Long	419	8.57 to 11.2	E	>200	711	5
**DDP** Didecyl phthalate	Long	447	9.05	F	>200	>1440	4
**DIDP** Diisodecyl phthalate	Long	447	10.36	F	>200	>1440	4

^a^ The iCSS ToxCast Dashboard (accessed on 20 May 2019), National Center for Biotechnology Information, PubChem Database (accessed on 20 May 2019), MW—molecular weight, log Kow—log value of n-octanol/water partition coefficient; ^b^_0.5h_EC_50_, the concentration causing a 50% decline in GJIC compared to the non-treated-cells after 0.5 h exposure; ^c^_80μM_ET_50_, the time causing a 50% decline in GJIC compared to the non-treated-cells after treatment to the concentration of 80 μM; ^d^ FOC, the fraction of the control (non-treated cells).

## Supplementary Materials

Supplementary materials can be found at https://www.mdpi.com/1422-0067/21/17/6069/s1.

## Abbreviations

BBP	Benzyl butyl phthalate
CaMgPBS	Calcium- and magnesium-supplemented PBS
CFDA-AM	5-Carboxyfluorescein diacetate, acetoxymethyl ester
DAP	Diallyl phthalate
DBP	Dibutyl phthalate
DCHP	Dicyclohexyl phthalate
DDP	Didecyl phthalate
DEHP	Di-(2-etylhexyl) phthalate
DEP	Diethyl phthalate
DHpP	Diheptyl phthalate
DIBP	Diisobutyl phthalate
DIDP	Diisodecyl phthalate
DIHpP	Diisoheptyl phthalate
DILI	Drug-induced liver injury
DINP	Diisononyl phthalate
DIPrP	Diisopropyl phthalate
DMP	Dimethyl phthalate
DOP	Dioctyl phthalate
DPeP	Dipentyl phthalate
DPhP	Diphenyl phthalate
DPrP	Dipropyl phthalate
GJIC	Gap junctional intercellular communication
LSPCs	Liver stem and progenitor cells
MAPK	Mitogen-activated protein kinases
MBP	Monobutyl phthalate
MEHP	Mono(2-ethylhexyl) phthalate
MINP	Monoisononyl phthalate
MMP	Monomethyl phthalate
MW	Molecular weight
NRU	Neutral red uptake
PPAR	Peroxisome proliferator-activated receptor
PBS	Phosphate buffered saline
SLDT	Scalpel load dye transfer
TPA	12-O-tetradecanoylphorbol 13-acetate
