# Supplementary material for: Structure-Dependent Effects of Phthalates on Intercellular and Intracellular Communication in Liver Oval Cells
Source: Int J Mol Sci. 2020 Aug 23;21(17):6069. doi: 10.3390/ijms21176069 (PMC7504421; doi:10.3390/ijms21176069)
Supplement: Supplementary file 1 [file ijms-21-06069-s001.pdf]

SUPPLEMENTARY INFORMATION

Structure-Dependent Effects of Phthalates on Intercellular and Intracellular Communication in Liver Oval Cells

Lucie Čtveráčková [1], Daniel Jančula [2], Jan Raška [1], Pavel Babica [1,2] and Iva Sovadinová [1,*]

[1] RECETOX, Faculty of Science, Masaryk University, Kamenice 753/5, Pavilion A29, 625 00 Brno, Czech Republic; lucie.ctverackova@recetox.muni.cz (L.Č.); raska@med.muni.cz (J.R.); pavel.babica@recetox.muni.cz (P.B.); iva.sovadinova@recetox.muni.cz (I.S.)

[2] Department of Experimental Phycology and Ecotoxicology, Institute of Botany of the Czech Academy of Sciences, Lidická 25/27, 602 00 Brno, Czech Republic; pavel.babica@ibot.cas.cz (P.B.)

* Correspondence: iva.sovadinova@recetox.muni.cz; Tel.: +420-549-494-738; Fax: +420-549-492-840

Table of Contents **Page No.**

TABLES

Table S1: Examples of national regulatory limits for phthalates 3

Table S2: Biological activity of studied phthalates concerning their hepatotoxicity 4-5

Table S3: The primers used in the study 6

FIGUES

Figure S1: Structure of studied phthalates 7

Figure S2: The scalpel cut in the confluent monolayer of WB F-344 cells exposed to phthalates 8

Figure S3: Cell viability of rat liver oval WB-F344 cells after 24-h treatment with phthalates evaluated by Alamar Blue® (AB) assay 9

Figure S4: Cell viability of rat liver oval WB-F344 cells after 24-h treatment with phthalates evaluated by neutral red uptake (NRU) assay 10

Figure S5: Lucifer yellow dye transfer in WB F-344 cells exposed to the phthalates reducing cell viability at least in one of the cytotoxicity assays 11

Figure S6: Original uncropped and unadjusted blot images for Figure 4 (Group A-C) – MAPK-pErk1/2 (A.) and GAPDH (B.) 12

Figure S7: Original uncropped and unadjusted blot images for Figure 4 (Group A-C) – MAPK-Erk1/2 (A.) and GAPDH (B.) 13

Figure S8: Original uncropped and unadjusted blot images for Figure 4 (Group D-F, TPA, DPhP) – MAPK-pErk1/2 (A.) and GAPDH (B.) 14

Figure S9: Original uncropped and unadjusted blot images for Figure 4 (Group D-F, TPA, DPhP) – total MAPK-Erk1/2 (A.) and GAPDH (B.) 15

Figure S10: Original uncropped and unadjusted gel images for Figure 5 – Actb and Pparα 16

Figure S11: Original uncropped and unadjusted gel images for Figure 5 –Pparβ/δ (A.) and Pparγ (B.) 17

Supplementary Tables

Supplementary Table S1: Examples of national regulatory limits for phthalates

Country	Phthalate	Regulation
Argentina[a]	DEHP, BBP, DBP, DOP, DINP	• Children's toys and daycare items for children <3 years old: DEHP + BBP + DBP <0.1%<br>• For articles that can be placed into a child's mouth: DEHP + BBP + DBP + DOP + DINP <0.1%
Australia[a]	DEHP	• Children's toys and daycare items for children <3 years old: <1%
Brazil[a]	DEHP, BBP, DBP, DOP, DINP	• Ethylvinyl toys and childcare articles: DEHP, BBP, DBP <1%<br>• Ethylvinyl toys and articles that can be placed in the mouth of children <3 years old: DEHP, BBP, DBP, DOP, DINP <1%
Canada[a]	DEHP, BBP, DBP	Ethyl vinyl toys and childcare articles: <0.1%
	DOP, DIDP, DINP	Soft ethyl vinyl toys and articles that can be placed in the mouth of children <4 years old: <0.1%
EU[a,b]	DEHP, DBP, BBP, DIBP	• Included in the list of REACH substances of very high concern, because of equivalent concern of "endocrine disrupting" properties in humans<br>• They have been restricted in the EU from 2019<br>• All children's toys or childcare articles for children <3 years old: DEHP + BBP + DBP <0.1%
	DCHP	• Included in the list of REACH substances of very high concern, because of equivalent concern of "endocrine disrupting" properties in human
	DINP, DOP	• All toys and childcare items that can be placed in a child's mouth: DIDP + DINP + DOP <0.1%
Japan[a,c]	DBP, DEHP, BBP	• <= 0.1% in the plasticized material in designated toys<br>• All synthetic polymer toys: DEHP is prohibited
	DIDP, DINP, DOP	• <=0.1% in the plasticized material in the parts that are intended to be placed in the mouth (excluding pacifiers and teething rings)<br>• For all mouth contact toys for children under 6 years old: DINP is prohibited<br>• DINP shall not be used as raw material for a toy that is intended to come in contact with the infant's mouth, PVC parts that are not intended to come in contact with the infant's mouth
USA[a,d]	DEHP, DBP, BBP	<=0.1% in children's toys and childcare articles
	DEHP, DBP, BBP, DINP, DIDP, DOP	<=0.1% in any children's toy that can be placed in a child's mouth and childcare articles

[a]Ashworth 2018, Int. J. Environ. Res. Public Health, 15, 200; [b] REACH Annex XVII restricted substances list (entry 51 and 52), [c] Japan Food Sanitation Law (Effective - September 6, 2011), [d] Consumer Product Safety Improvement Act of 2008 (CPSIA). **BBP**, Benzyl butyl phthalate; **DBP**, Dibutyl phthalate; **DCHP**, dicyclohexyl phthalate; **DEHP**, Di-(2-ethyl hexyl) phthalate; **DIBP**, Diisobutyl phthalate; **DIDP**, Diisodecyl phthalate; **DINP**, Diisononyl phthalate; **DOP**, Dioctyl phthalate.

Supplementary Table S2: Biological activity of studied phthalates concerning their liver toxicity

Phthalate		Literature search					ToxCast activity [c]										
		Hazard traits	*In vivo* & *In vitro* assays concerning hepatic cells [b]				No. of ToxCast assays tested	% positive hits	Active assays per gene set								Active assays
		Liver tox./ tumor [a]	PPARα	PPARß/δ	PPARγ	GJIC			PPARs	CAR	PXR	LXR	RXR	FXR	AhR	CYPs	Hepatotox.
Group A	**MMP**		-	-	-		1084	1.38	0	0	1	0	0	1	0	1	3
	**DMP**	+/-	-	-	-		1081	1.11	0	0	1	0	1	0	0	0	3
	**DEP**	+/+	-	-	-		881	1.14	0	0	0	0	0	0	0	0	1
	**MBP**		+/-	+/-	+/-		1080	1.57	1	0	2	0	0	0	1	1	6
Group B	**DPrP**						513	4.09	2	0	2	0	1	0	0	0	9
	**DIPrP**		-	-	+		423	4.09	1	0	1	1	2	0	1	0	13
	**DAP**	+/-	+	+	+		882	6.8	2	0	2	2	2	0	0	4	21
Group C	**DBP**	+/	+	+	+		1087	7.33	1	1	2	0	1	1	0	11	22
	**DIBP**	+/-	+		+		878	11.39	2	0	1	1	1	1	0	1	36
	**BBP**	+/-	+	+	+		882	7.82	2	0	1	0	0	2	0	2	12
	**DPeP**	+/	+	+	+		883	8.49	1	0	2	1	1	1	0	0	21
	**DCHP**	+/-					608	26.97	2	0	2	0	2	3	1	0	22
	**DPhP**						560	11.61	2	0	2	1	0	0	0	0	8
Group D	**DHpP**						450	1.78	1	1	2	0	0	0	0	0	4
	**DIHpP**					-	211	2.84	0	0	0	0	0	0	0	0	3
Group E	**DOP**	+/			+	-	885	5.08	1	0	1	0	0	0	0	3	3
	**DEHP**	+/+	+/-	-	+/-	+/-	1080	4.17	2	0	2	0	0	0	0	2	8
	**DINP**	+/+	+		+	+/-	553	2.13	0	0	2	0	0	0	0	0	3
Group F	**DDP**						211	0.95	1	0	0	0	0	0	0	0	0
	**DIDP**	+/				-	552	1.27	0	0	1	0	3	0	0	0	2

[a] A "+" or "-" notation indicates data from *in vivo* studies in animals or *in vitro* studies are available to support or not to support, respectively, chemical activity for the hazard trait - Pham 2016, Tox Sci 151, 286; the Chemistry Dashboard, https://comptox.epa.gov/dashboard/ (accessed 5/2019); [b] A "+" or "-" notation indicates data from *in vivo* studies in animals or *in vitro* studies are available to support or not support, respectively, chemical interaction with the appropriate molecular target - Bility 2004, Tox Sci 82, 170; Corton 2005, Tox Sci 83, 4; Hurst 2003, Tox Sci 74, 297; Isenberg, 2000, Tox Sci 56, 73; Kanyama 2005, Mol Pharmacol 67, 766; Lampen 2003, Toxicol Appl Pharmacol 188, 14; Lapinskas 2005, Toxicology 207, 149; Maloney 1999, Toxicol Appl Pharmacol 161, 209; McKee, 2000, Regul Toxicol Pharmacol 32, 51; The Office of Environmental Health Hazard Assessment's (OEHHA), 2013, https://oehha.ca.gov/media/downloads/proposition-65/chemicals/dinphid100413.pdf; Pham 2016, Tox Sci 151, 286; Pugh 2000, Tox Sci 56, 181; Smith 2000, Tox Sci 54, 312; Valles 2003, Toxicology 191, 211; [c] The number indicates the number of assay endpoints in each gene set concerning hepatoxicity or hepatic tumors or in the hepatotoxicity assays with rat and human hepatocytes or HepG2 cells - Pham 2016, Tox Sci 151, 286; ToxCast dashboard (https://actor .epa.gov/dashboard/), the Chemistry Dashboard, https://comptox.epa.gov/dashboard/ (both accessed 5/2019). Blank cells indicate a lack of data. **AhR**,aryl hydrocarbon receptor; **CAR**, constitutive androstane receptor; **CYPs**, cytochromes P450; **FXR**, farnesoid X receptor; **GJIC**, gap junctional intercellular communication; **LXR**, liver X receptor; **PPARs**, peroxisome proliferator-activated receptors; **PXR**, pregnane X receptor; **RXR**, retinoid X receptor.

Supplementary Table S3: The primers used in the study

Gene	Accession No.	Primer sequences (F - forward, R - Reverse)	Splice variants	Product size (bp)	Source
Pparα	NM_013196.1	F - gatgacagtgacatttcc<br>R - gaagagaaaggtatcatcc	NM_013196.1	159	Primer 3
Pparß/δ	NM_013141.2	F - caacaagtgtcagtactgc<br>R - attgtagatgtgcttgg	NM_013141.2	187	Primer 3
Pparγ	NM_001145367.1	F – ctcatacataaagtccttcc<br>R - atactctgtgatctcttgc	NM_001145367.1<br>NM_013124.3<br>NM_001145366.1	238	Primer 3
ß-Actin	NM_007393.5	F – aaccttcttgcagctcctcc<br>R - ccatacccaccatcacaccc	NM_007393.5	193	Primer 3

SUPPLEMENTARY FIGURES

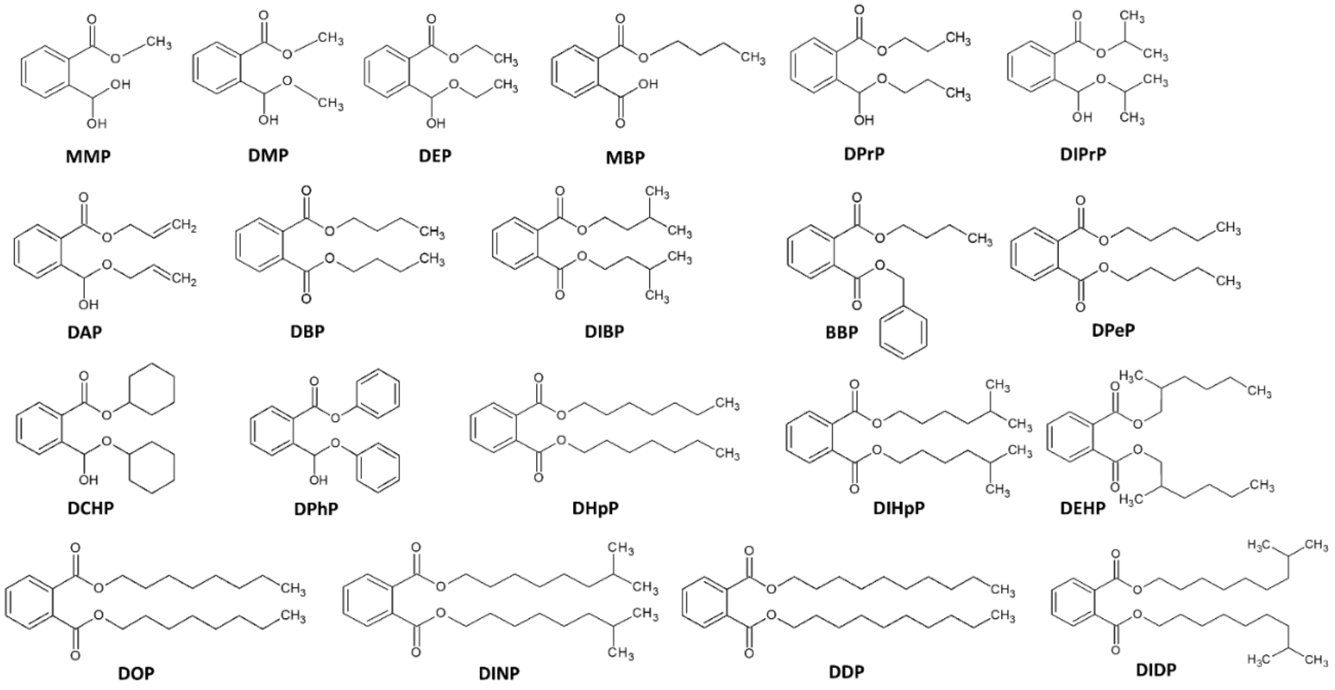

Supplementary Figure S1: Structure of studied phthalates.

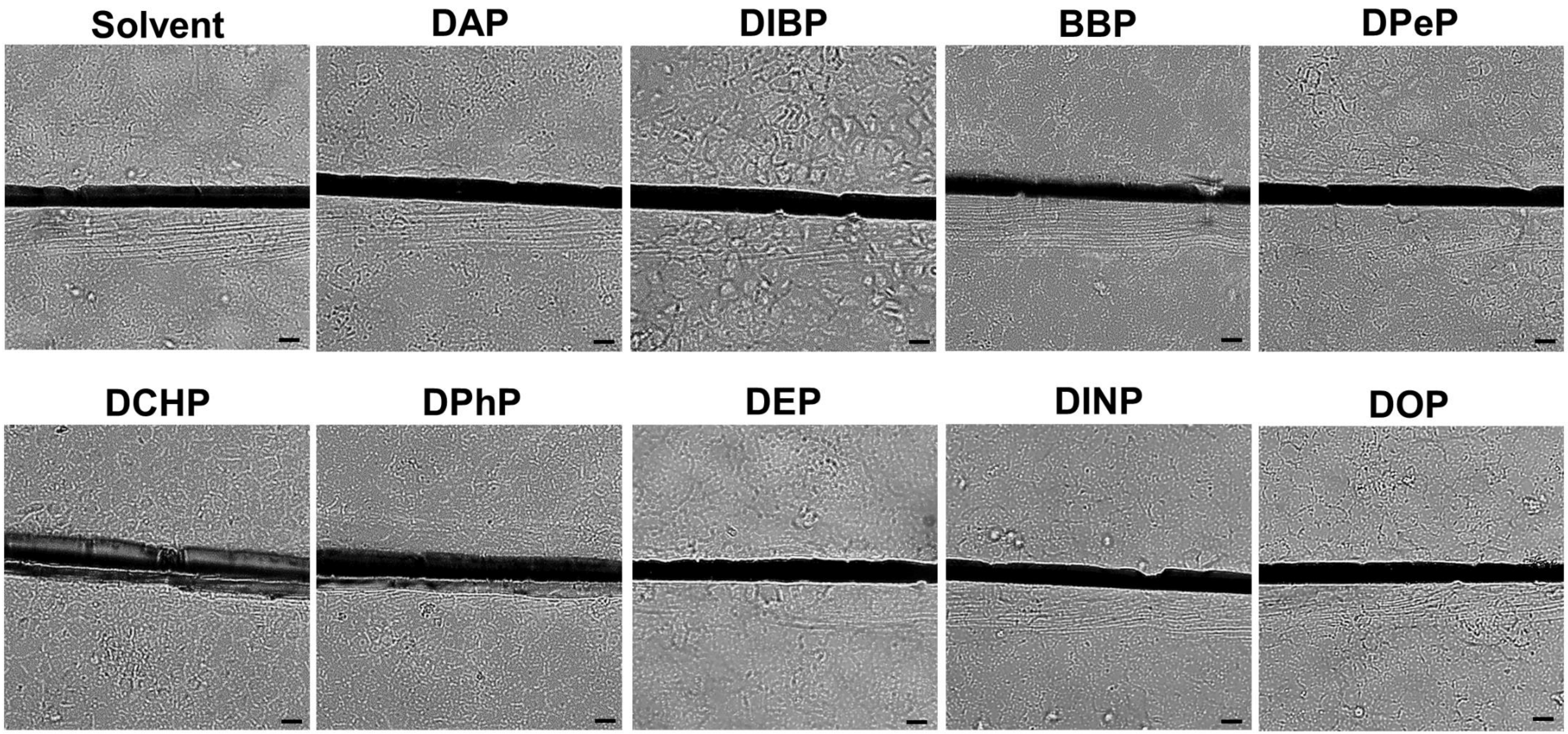

Supplementary Figure S2: The scalpel cut in the 100% confluent monolayer of WB F-344 cells exposed to phthalates. The representative bright-field images of the cut after 24-h exposure of cells to phthalates at the highest concentrations tested in GJIC assay (80 μM) followed by the SL-DT technique. The phthalates did not disturb the cell confluent monolayer (no gaps between cells or a large number of detached cells floating). Scale bar = 50 μm. **BBP**, benzyl butyl phthalate; **DAP**, diallyl phthalate; **DCHP**, dicyclohexyl phthalate; **DEP**, diethyl phthalate; **DIBP**, diisobutyl phthalate; **DINP**, diisononyl phthalate; **DOP,** dioctyl phthalate; **DPeP**, dipentyl phthalate; **DPhP**, diphenyl phthalate.

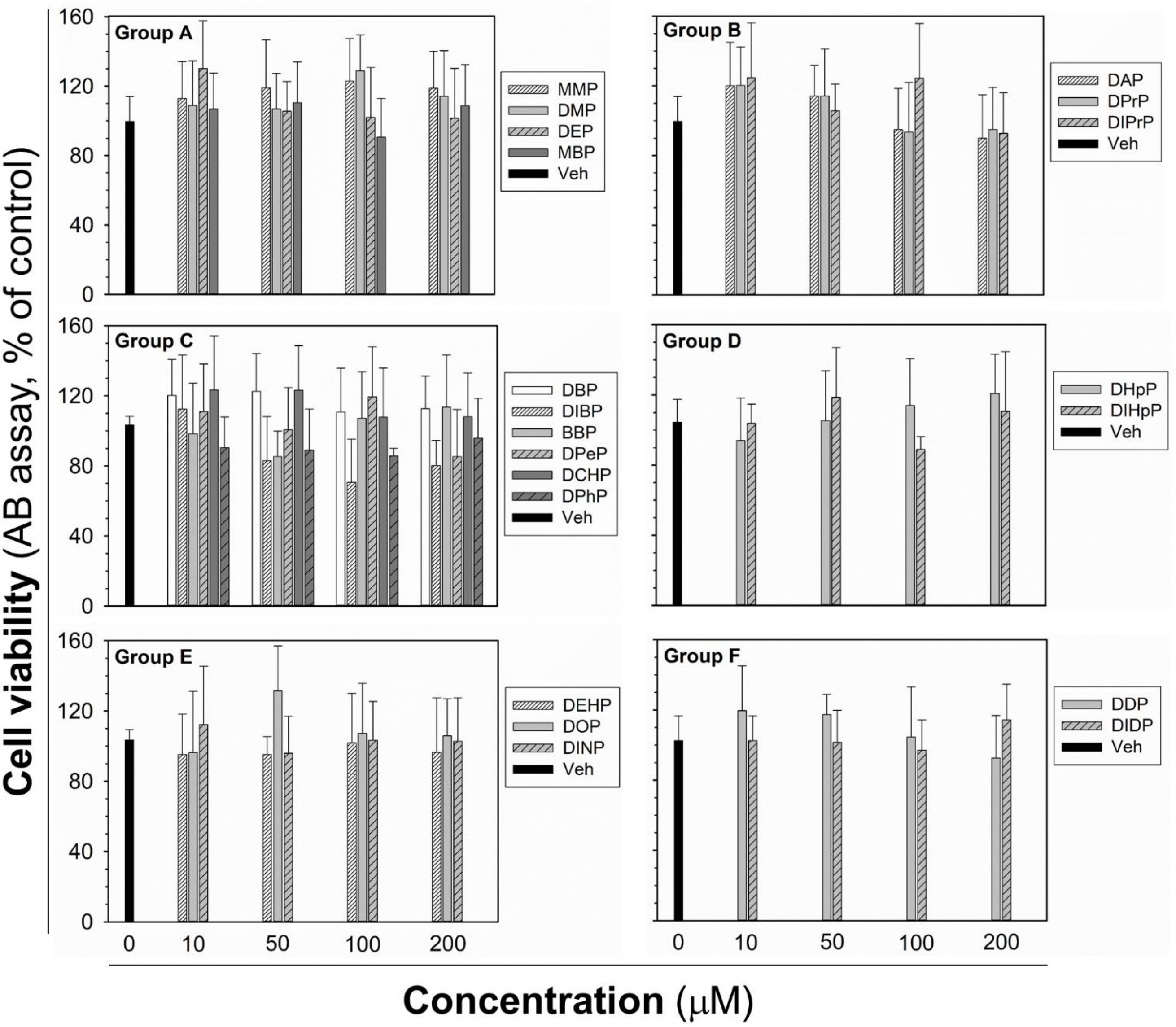

Supplementary Figure S3: Cell viability of rat liver oval WB-F344 cells after 24-h treatment with phthalates evaluated by Alamar Blue® (AB) assay. Data represent means (SD) of independent experiments (n>3). Significant differences from the vehicle control were determined by one-way ANOVA/Kruskal-Wallis ANOVA (*ns*, P > 0.050).

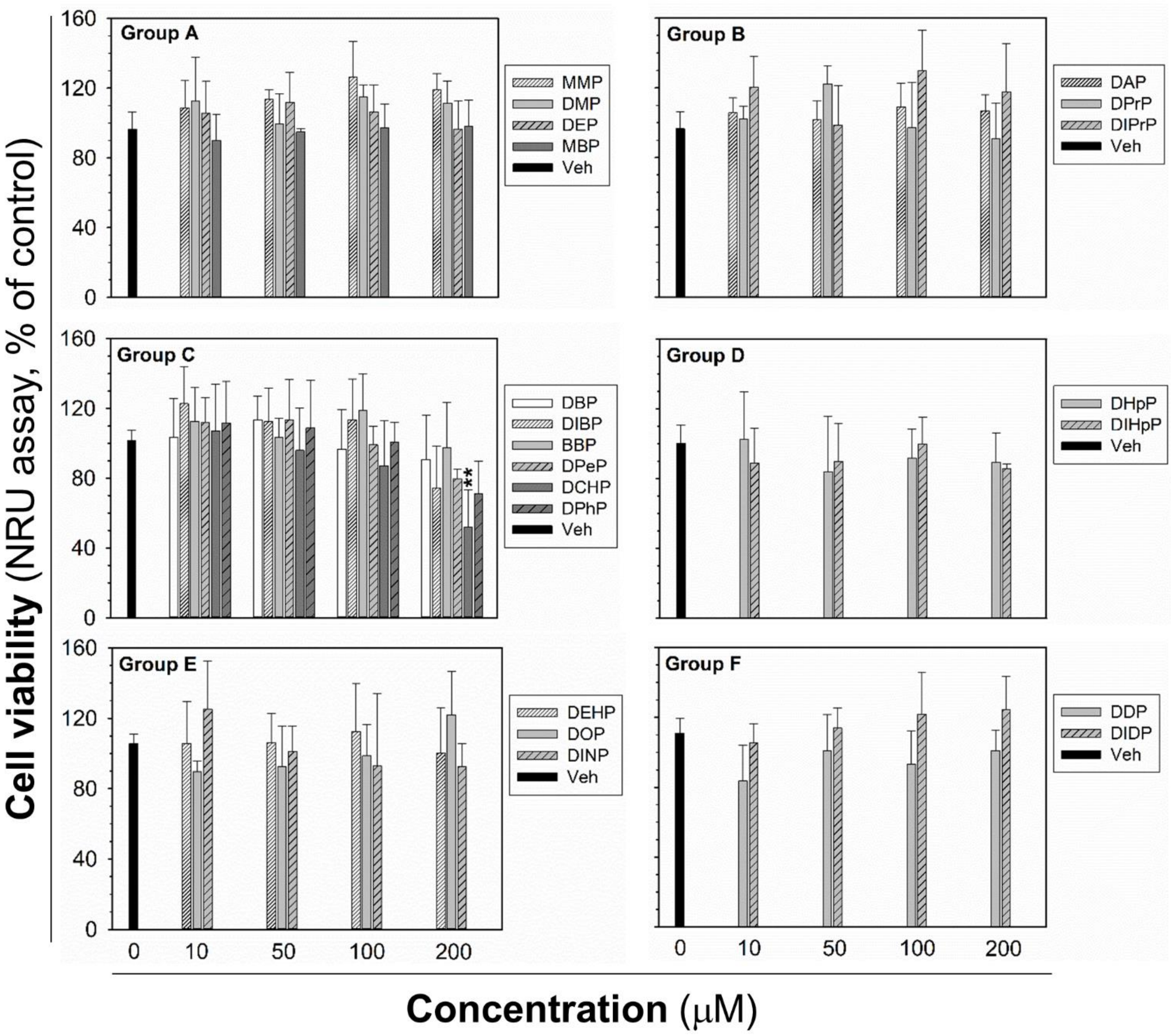

Supplementary Figure S4: Cell viability of rat liver oval WB-F344 cells after 24-h treatment with phthalates evaluated by neutral red uptake (NRU) assay. Data represent means (SD) of independent experiments (n>3). Significant differences from the vehicle control were determined by one-way ANOVA followed by Dunnett's post hoc test (**, P ≤ 0.010).

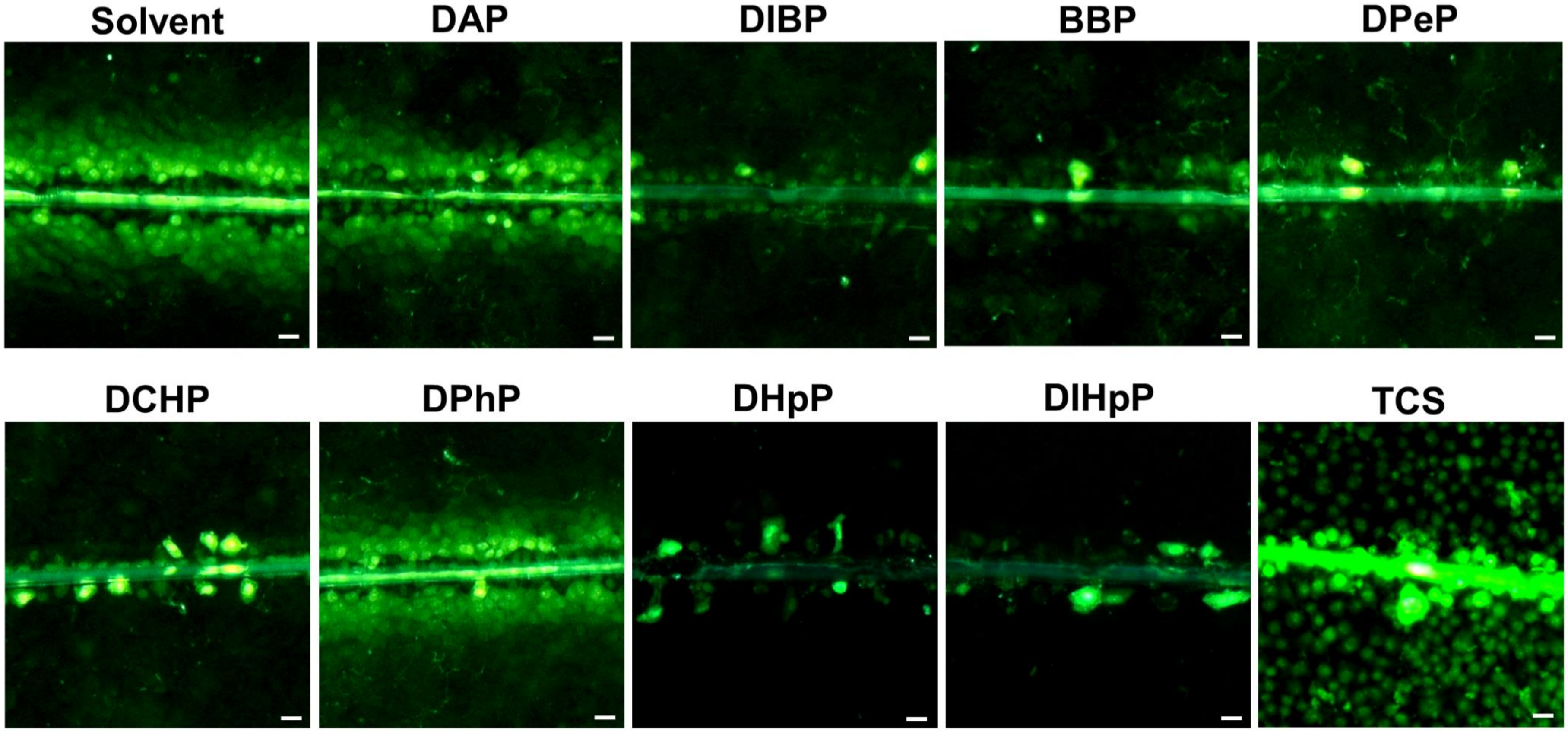

Supplementary Figure S5: Lucifer yellow dye transfer in WB F-344 cells exposed to the phthalates found to reduce cell viability in at least one of the cytotoxicity assays. The representative fluorescent images after 24-h exposure of cells to phthalates at the highest concentrations tested in GJIC assay (80 μM) followed by the SL-DT technique. The lucifer yellow dye spreading into the WB F-344 cells is related to the GJIC extent. Non-specific spreading of lucifer yellow (higher background) due to possible cytotoxicity of phthalates was not observed, which is in contrast to the effect of triclosan (TCS) at the cytotoxic concentration of 100 μM. Scale bar = 50 μm. **BBP**, benzyl butyl phthalate; **DAP**, diallyl phthalate; **DCHP**, dicyclohexyl phthalate; **DHpP**, diheptyl phthalate; **DIBP**, diisobutyl phthalate; **DIHpP**, diisoheptyl phthalate; **DPeP**, dipentyl phthalate; **DPhP**, diphenyl phthalate; **TCS**, triclosan.

A.

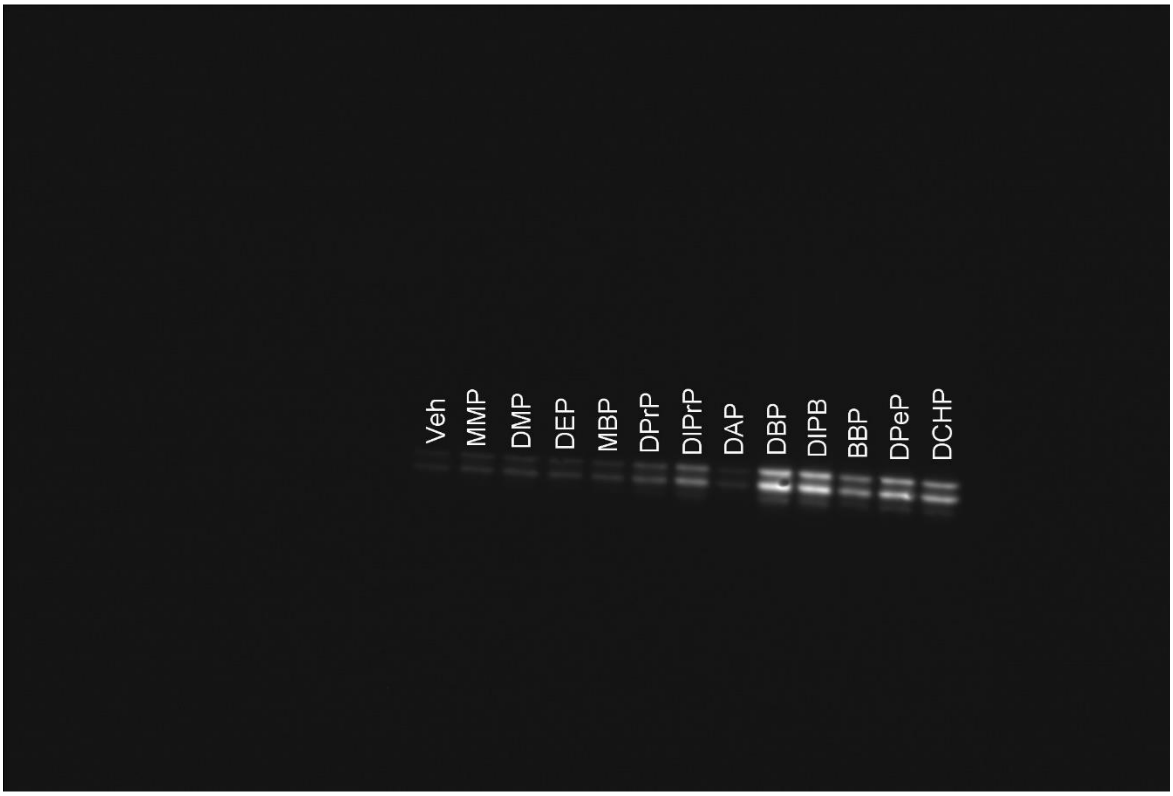

B.

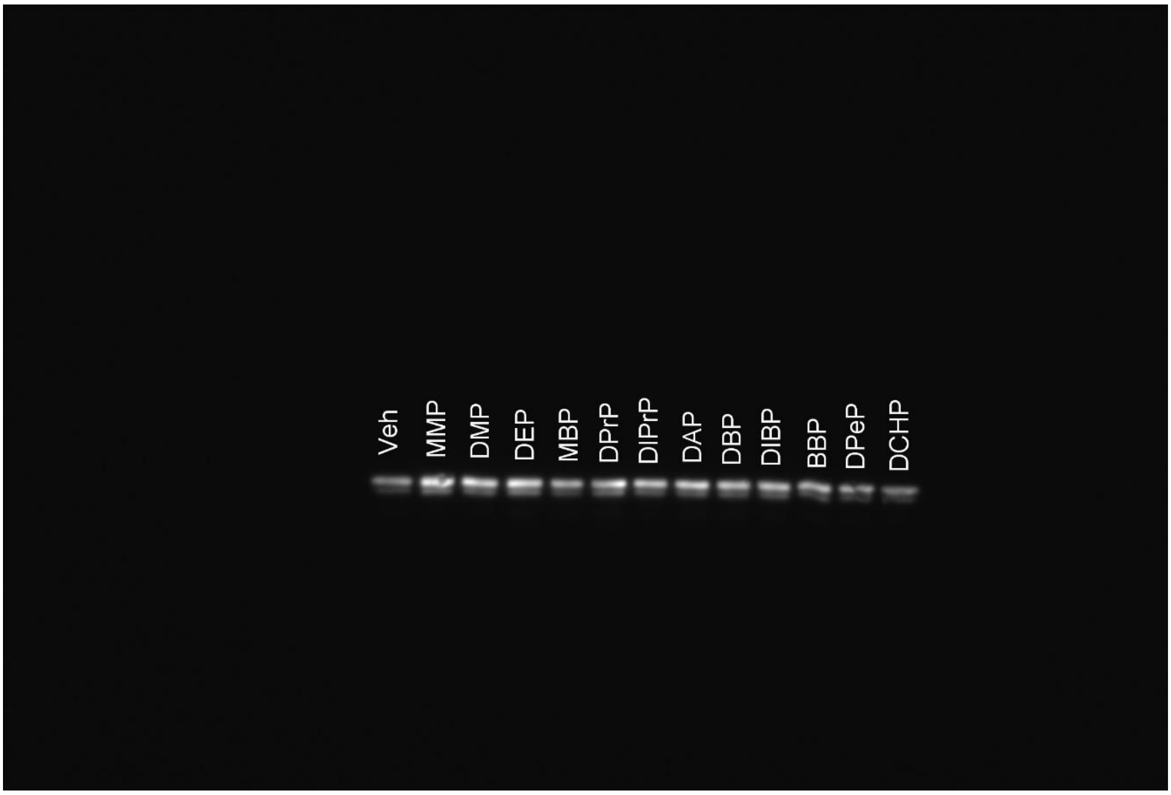

Supplementary Figure S6: Original uncropped and unadjusted blot images for Figure 4 (Group A-C) – MAPK-pErk1/2 (A.) and GAPDH (B.)

A.

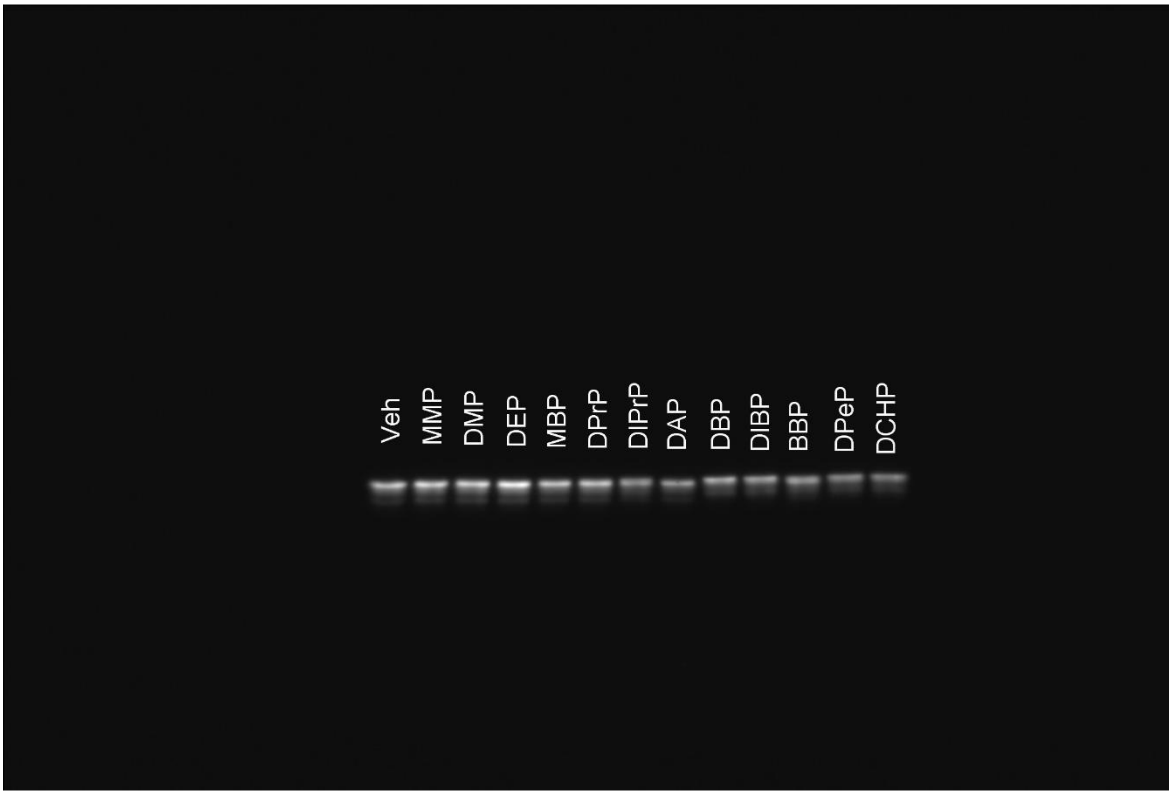

B.

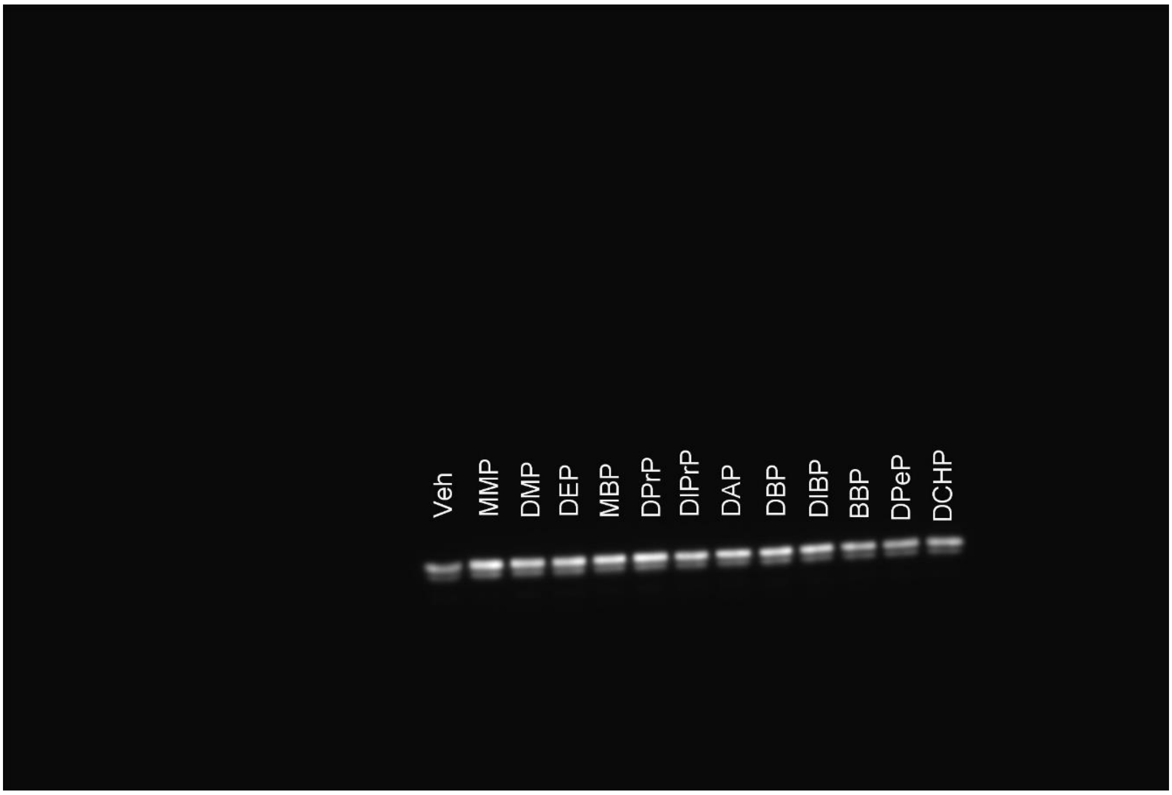

Supplementary Figure S7: Original uncropped and unadjusted blot images for Figure 4 (Group A-C) – total MAPK-Erk1/2 (A.) and GAPDH (B.)

A.

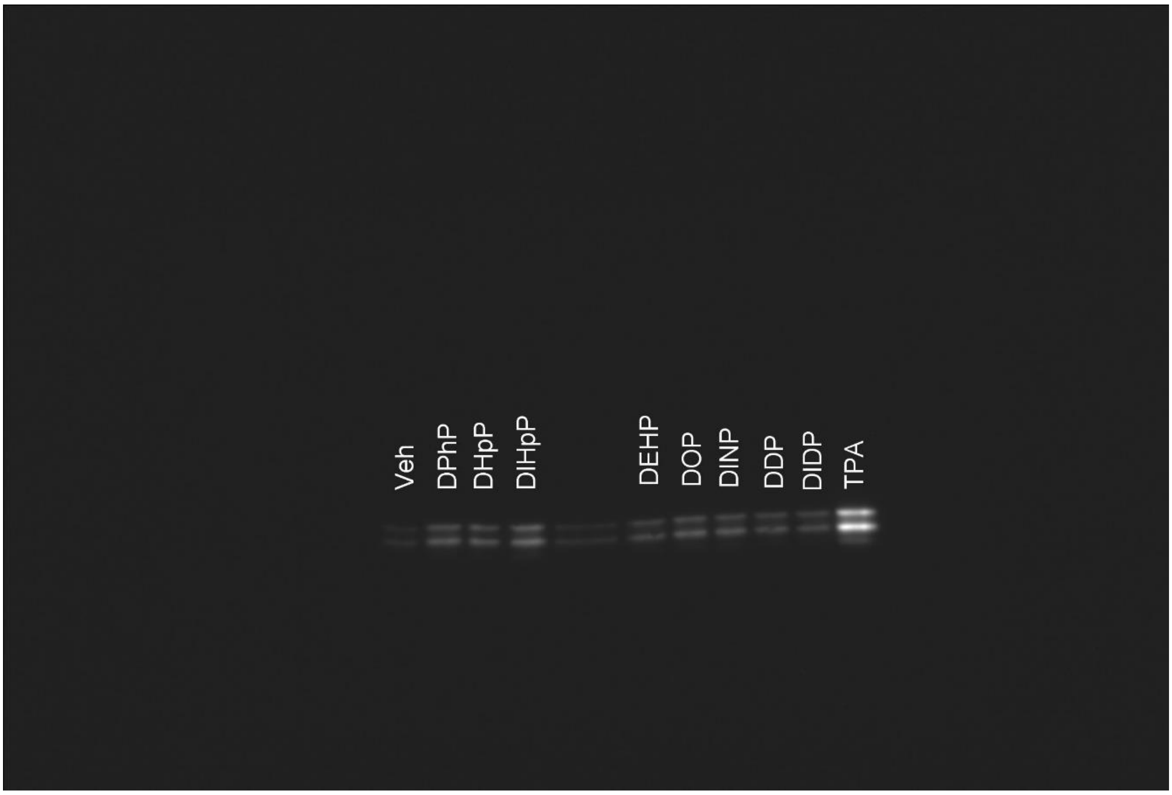

B.

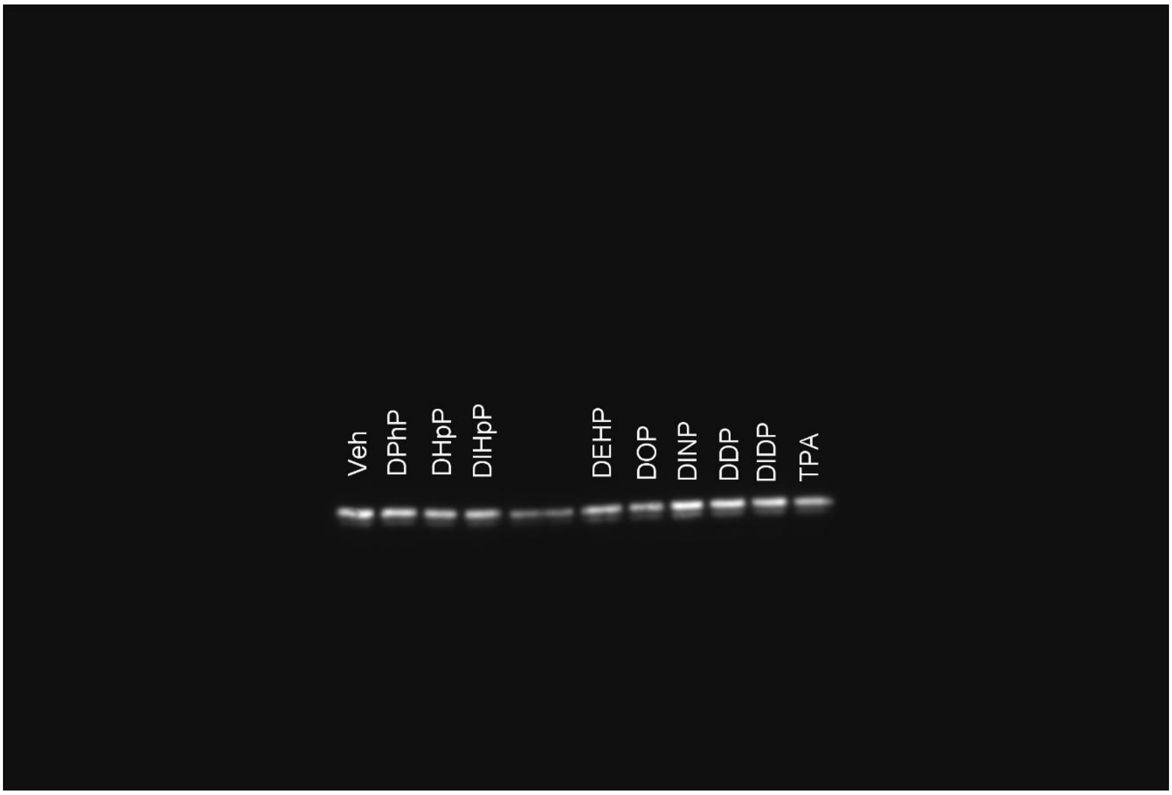

Supplementary Figure S8: Original uncropped and unadjusted blot images for Figure 4 (Group D-F, TPA, DPhP) – MAPK-pErk1/2 (A.) and GAPDH (B.)

A.

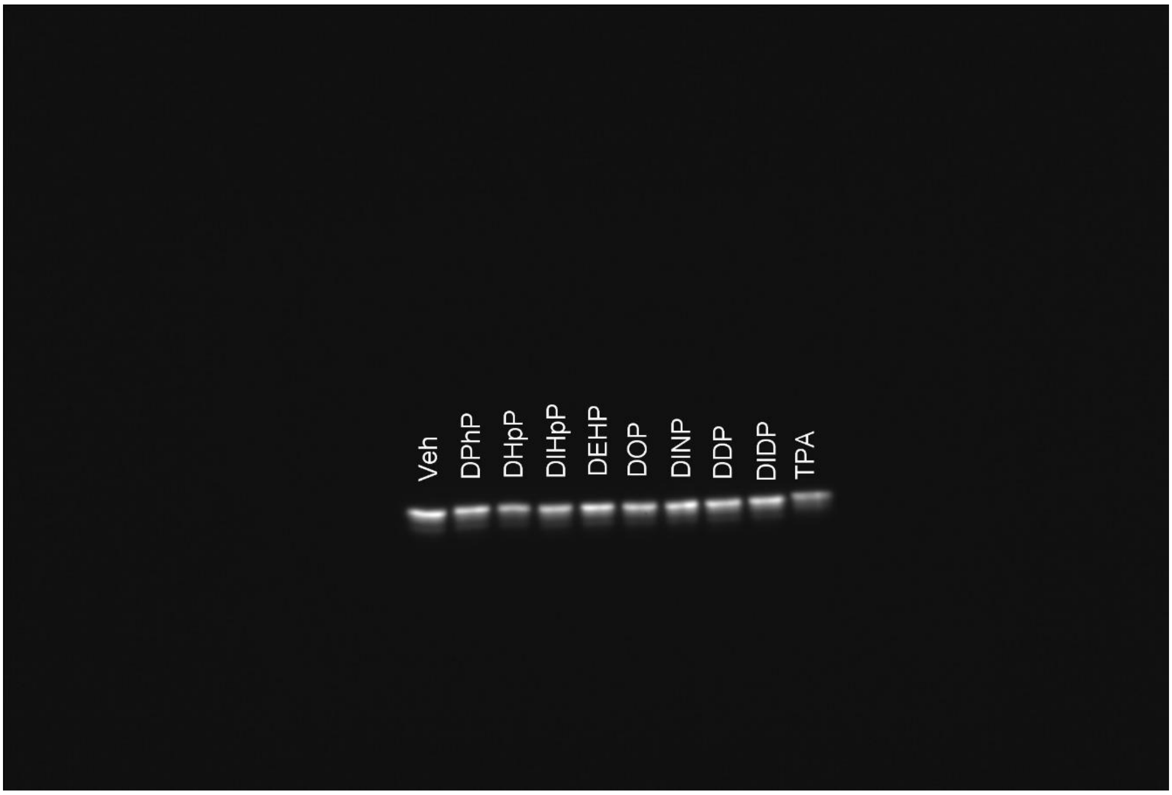

B.

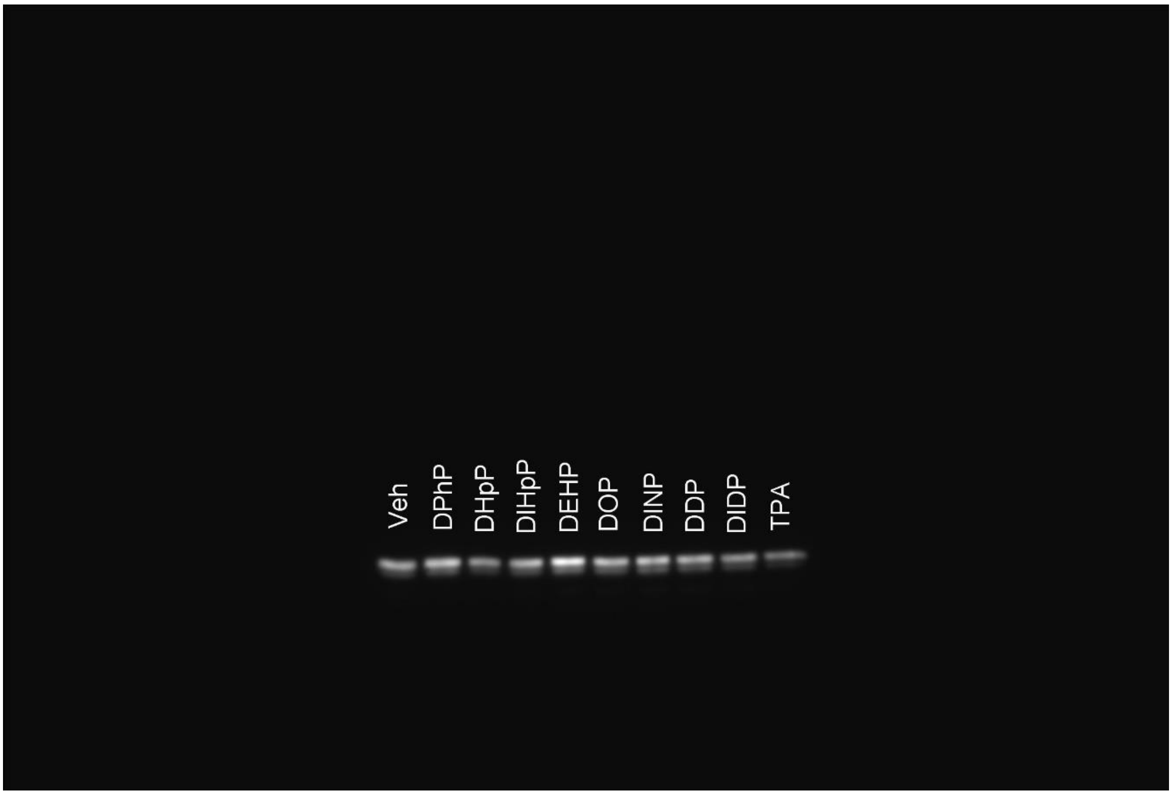

Supplementary Figure S9: Original uncropped and unadjusted blot images for Figure 4 (Group D-F, TPA, DPhP) – total Erk1/2 (A.) and GAPDH (B.)

A.

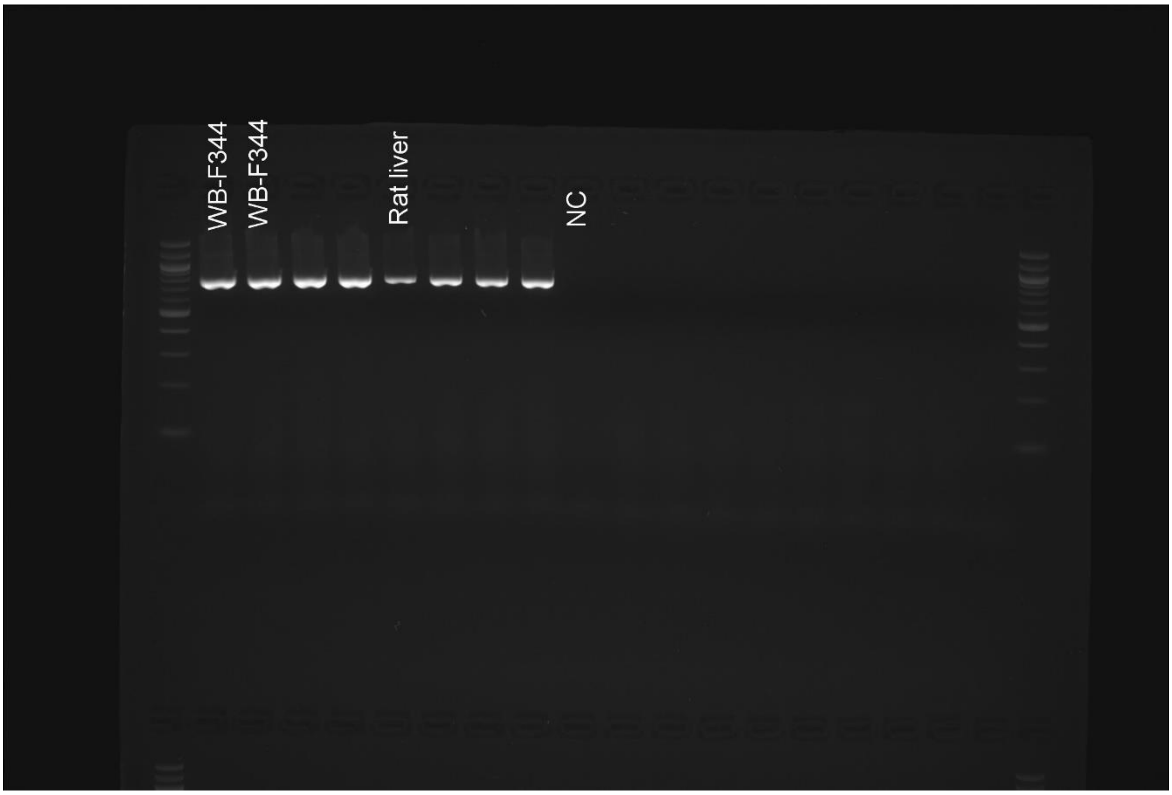

B.

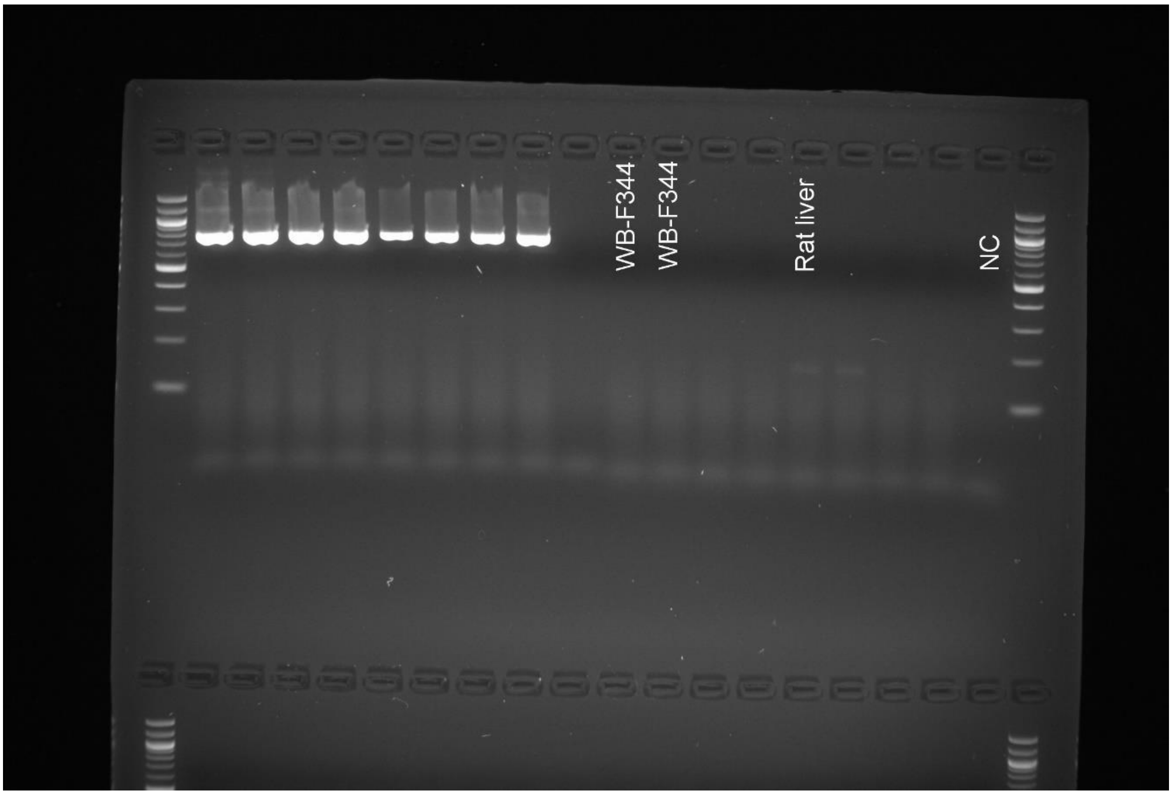

Supplementary Figure S10: Original uncropped and unadjusted gel images for Figure 5 – *Actb* (A.) and *Pparα (B.)*

A.

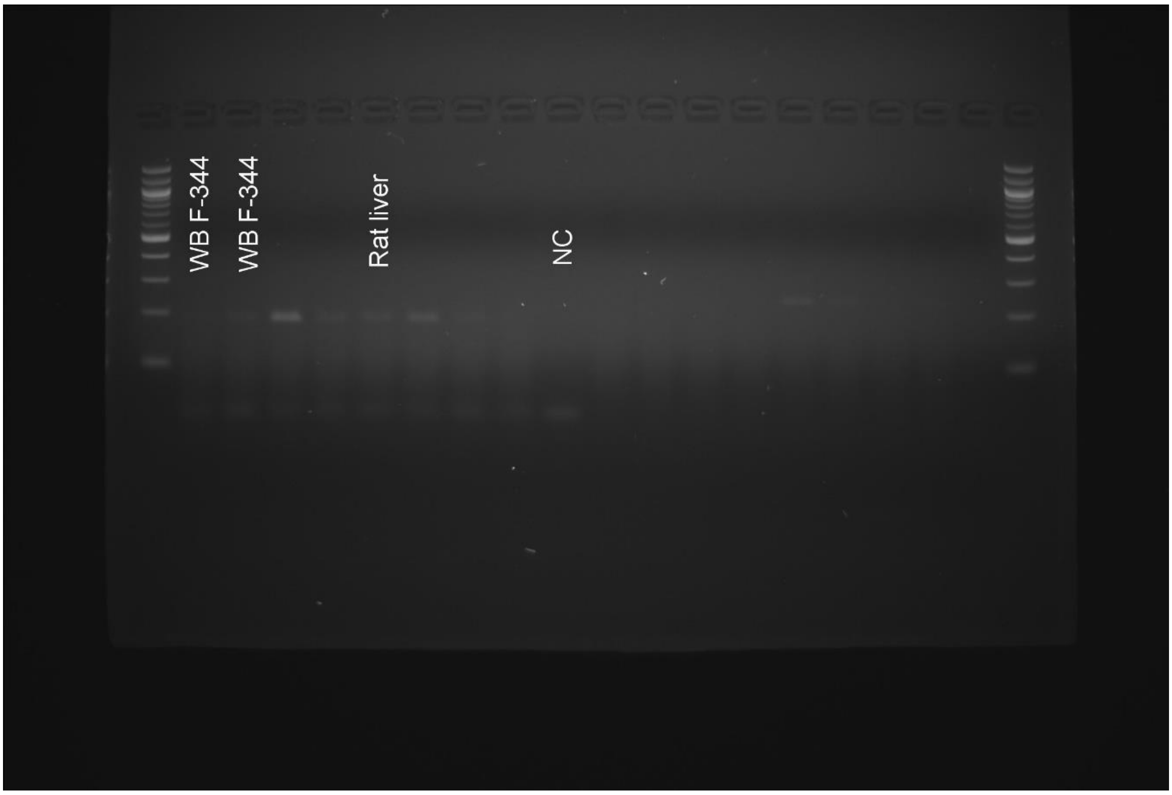

B.

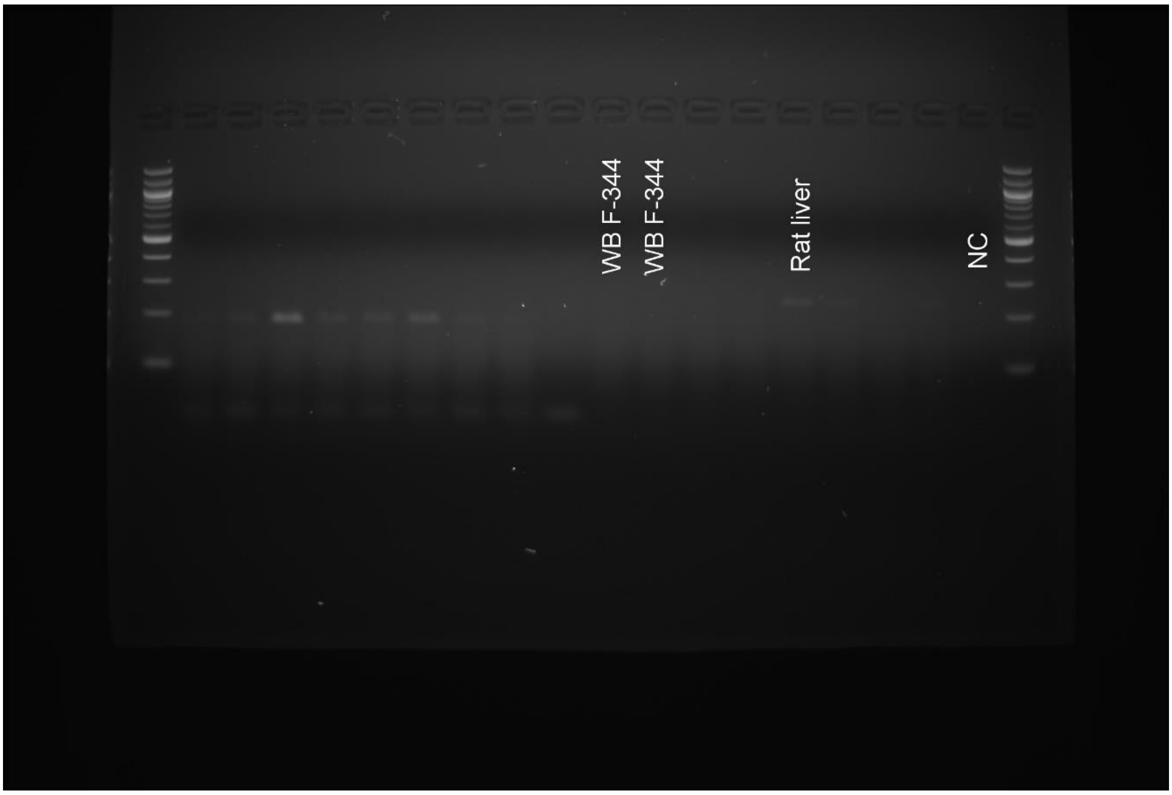

Supplementary Figure S11: Original uncropped and unadjusted gel images for Figure 5 –*Pparβ/δ* (A.) and *Pparγ* *(B.)*
